# High-fat diet induces depression-like phenotype via astrocyte-mediated hyperactivation of ventral hippocampal glutamatergic afferents to the nucleus accumbens

**DOI:** 10.1038/s41380-022-01787-1

**Published:** 2022-09-30

**Authors:** Sheng-Feng Tsai, Pei-Ling Hsu, Yun-Wen Chen, Mohammad Shahadat Hossain, Pei-Chun Chen, Shun-Fen Tzeng, Po-See Chen, Yu-Min Kuo

**Affiliations:** 1 Institute of Basic Medical Sciences, College of Medicine, National Cheng Kung University, Tainan, 70101 Taiwan, ROC; 2 Department of Cell Biology and Anatomy, College of Medicine, National Cheng Kung University, Tainan, 70101 Taiwan, ROC; 3 Department of Physiology, College of Medicine, National Cheng Kung University, Tainan, 70101 Taiwan, ROC; 4 Department of Anatomy, School of Medicine, College of Medicine, Kaohsiung Medical University, Kaohsiung, 80708 Taiwan, ROC; 5 Department of Pharmacology, College of Medicine, National Cheng Kung University, Tainan, 70101 Taiwan, ROC; 6 Interdisciplinary Neuroscience, Taiwan International Graduate Program, Academia Sinica, National Cheng Kung University, Tainan, 70101 Taiwan, ROC; 7 Department of Life Sciences, College of Bioscience and Biotechnology, National Cheng Kung University, Tainan, 70101 Taiwan, ROC; 8 Department of Psychiatry, College of Medicine, National Cheng Kung University, Tainan, 70101 Taiwan, ROC; 9 Addiction Research Center, National Cheng Kung University Hospital, National Cheng Kung University, Tainan, 70403 Taiwan, ROC

**Keywords:** Depression, Neuroscience

## Abstract

Comorbidity exists between metabolic disorders and depressive syndrome with unclear mechanisms. To characterize the causal relationship, we adopted a 12-week high-fat diet (HFD) to induce metabolic disorder and depressive phenotypes in mice. Initially, we identified an enhanced glutamatergic input in the nucleus accumbens of HFD mice. Retrograde tracing and chemogenetic inhibition showed that the hyperactive ventral hippocampal glutamatergic afferents to the nucleus accumbens determined the exhibition of depression-like behavior in HFD mice. Using lentiviral knockdown and overexpression approaches, we proved that HFD-induced downregulation of glial glutamate transporters, GLAST and GLT-1, contributed to the observed circuit maladaptations and subsequent depression-like behaviors. Finally, we identified a potential therapeutic agent, riluzole, which could mitigate the HFD-induced behavioral deficits by normalizing the expressions of GLAST and GLT-1 and ventral hippocampal glutamatergic afferents to the nucleus accumbens. Overall, astrocyte-mediated disturbance in glutamatergic transmission underlies the metabolic disorder-related depressive syndrome and represents a therapeutic target for this subtype of depressive mood disorders.

## Introduction

Depressive mood disorder is a common, debilitating disorder, frequently comorbid with many chronic diseases, more often with metabolic disorders (MetD) and cardiovascular diseases [1–6]. Depressive syndrome and MetD are thought to bidirectionally affect each other. For example, social defeat stress, a paradigm widely used to induce a depressive phenotype, potentiates systemic insulin resistance in mice with diet-induced obesity [7]. Moreover, some antidepressants have been shown to improve glycemic control in adults with comorbid depression and type 2 diabetes, while some antidiabetic agents may show benefits for depression phenotypes [8–11]. The major underlying risk factors for MetD are abdominal obesity and insulin resistance [12]. Moreover, depression-like behavior has been reported in a mouse model of obesity/MetD induced by long-term consumption of high-fat diet (HFD) [13–16]. Although insulin resistance, inflammation, and hyperactivity of the hypothalamic-pituitary-adrenal axis have been suggested to mediate the phenotypic overlap of MetD and depressive syndrome [4, 17], the precise molecular mechanism and neural substance underlying MetD-related depressive syndrome remains elusive.

Perturbation of the mesolimbic dopaminergic (DA) reward circuit, comprising DA neurons in the ventral tegmental area (VTA) and GABAergic neurons in the nucleus accumbens (NAc), is one of the main focuses in the investigation of the neural pathophysiology of depression [18–20]. Hyperactivation of DA neurons that project from the VTA to NAc (VTA→NAc) is a hallmark of depressive mice susceptible to chronic social defeat stress (CSDS) [21–25]. The activity of VTA DA neurons can be regulated by glutamatergic afferents to the NAc since a direct infusion of glutamate into the NAc induces a depressive phenotype [26]. The medial prefrontal cortex (mPFC), basolateral amygdala (BLA), and ventral hippocampus (vHPC) represent the three major glutamatergic NAc-projecting centers and are associated with the motivation, processing of fear and harmful information, and the integration in the development of depression [27–30]. Whether the NAc-projecting glutamatergic neurons are hyperactive in MetD-related depressive syndrome remains unclear.

Herein, we adopted the HFD-induced obesity mouse model to investigate the mechanism underlying MetD-related depressive syndrome. We examined the glutamatergic NAc-projecting center activities to search for brain region(s) and glutamate transmission-related molecules involved in exhibiting MetD-related depressive syndrome.

## Materials/subjects and methods

### Animals

Animal experiments were approved by the National Cheng Kung University Institutional Animal Care and Use Committee (approval number: 104243, 106057, and 109139) and were in accordance with local and national guidelines. Mice were obtained from and maintained at National Cheng Kung University Laboratory Animal Center (NCKULAC, Tainan, Taiwan) accredited by AAALAC. The housing conditions and detailed number of animals and treatment timelines for each experiment are described in Supplementary 1 and Supplementary Table S1.

### Experimental model and designs

Male C57BL/6N mice (8-week-old) were randomly assigned to regular chow diet (CD) and HFD groups with computer-based randomization. HFD mice were fed with commercial HFD (Cat# 58Y1, TestDiet, St. Louis, MO, USA) for 12 weeks to induce obesity, systemic insulin resistance, and depressive phenotypes. Five cohorts of mice were subjected to the following studies: 1) examination of the effects of HFD on the exhibition of depression-like behaviors and activities of glutamatergic afferents to the NAc; 2) identification of the neural circuit that determines the depression-like behavior in HFD mice using the chemogenetic approach; 3 and 4) clarifications of the role of glial glutamate transporters in the developments of circuit maladaptations and behavioral deficits by lentiviral knockdown and overexpression approaches; 5) examination of the therapeutic effect of glutamate modulator, riluzole (RLZ), on HFD-induced depression-like behaviors.

### Behavior tests

Sucrose preference test (SPT) and forced swimming test (FST) were performed to evaluate depression-like behaviors in mice 1 day after the conclusion of HFD and/or treatment. The detailed protocols are described in Supplementary 1.

### Assessments of glutamatergic activity afferents to the NAc

The extracellular glutamate levels in region of interests were measured by real-time intra-cranial biosensors. The NAc-projecting glutamatergic neurons were retrogradely labeled by FluoroGold (FG, Cat# sc-358883, Santa Cruz Biotechnology, Dallas, TX, USA) tracer or adeno-associated viruses with a retrograde capsid (Cat# 50475-AAVrg and114472-AAVrg, Addgene, Watertown, MA, USA). Their activities were determined by c-Fos expression. The detailed methods are described in Supplementary 1.

### Chemogenetic inhibition

The chemogenetic inhibitory approach was used to silence NAc-projecting vHPC glutamatergic neurons and determine the effects of this circuit on FST performance. Six weeks before ending HFD, the rAAV expressing the engineered human M4 muscarinic receptor couples with Gi protein (hM4D_Gi_) and mCherry (titer ≥ 7 × 10¹² vg/mL; pAAV-hSyn-hM4D_Gi_-mCherry, Cat# 50475-AAVrg, Addgene) was bilaterally infused into the NAc of mice. On the experiment day, CNO (Cat# 4936, Tocris Bioscience, Bristol, UK) was bilaterally infused into the vHPC (2 µg/µL dissolved in saline, 1 µL/side) of free-moving mice, 30 min before the depression-like behavioral test, to transiently inhibit neuronal activity [31]. Mice were sacrificed 120 min after the completion of CNO infusions. Those mice that received bilateral infusions of an equal volume of saline to the vHPC served as control. Please see Supplementary 1 for detailed protocols of rAAV production and infusion.

### Lentiviral knockdown and overexpression

Lentiviruses (LVs) expressing short hairpin RNA against GLAST and GLT-1 or expressing GLAST and GLT-1 were bilaterally infused into the vHPC of mice 4 weeks before concluding the HFD. Please see Supplementary 1 for detailed protocols of LV production and infusion.

### RLZ treatment in mice

We tested the therapeutic effects of RLZ administered via both systemic and intracranial routes. In the systemic administration experiment, mice of both CD and HFD groups were injected daily with RLZ (Cat# 1604337, Sigma-Aldrich, St. Louis, MO, USA; 4 mg/kg dissolved in normal saline containing 1% DMSO, i.p.) for the last 3 weeks of the 12-week HFD period. Other groups of CD and HFD mice received daily infusions of vehicle (equal volume of saline containing 1% DMSO) served as vehicle controls. In the intracranial administration experiment, mice of both CD and HFD groups were bilaterally implanted with cannulas into the vHPC 10 days before the end of the 12-week HFD feeding period. After a 3-day recovery period, the mice received daily infusions of RLZ (1 nmol/side, dissolved in 0.5 µL of artificial cerebrospinal fluid containing 1% DMSO; infusion rate: 0.05 µL/min) for 7 days. Other groups of CD and HFD mice received daily infusions of vehicle (equal volume of artificial cerebrospinal fluid containing 1% DMSO) served as vehicle controls.

### Statistical analysis

All numerical data are expressed as mean ± standard deviation. Statistical analyses and graph plotting were performed using the Prism software (v. 7.0a, GraphPad Software Inc., San Diego, CA, USA). Significance was set at *p* < 0.05. The details of statistical analysis and results are described in Supplementary 1 and Supplementary Table S2.

## Results

### HFD induces depression-like behaviors and enhances NAc glutamatergic inputs in mice

**Fig. 1:**
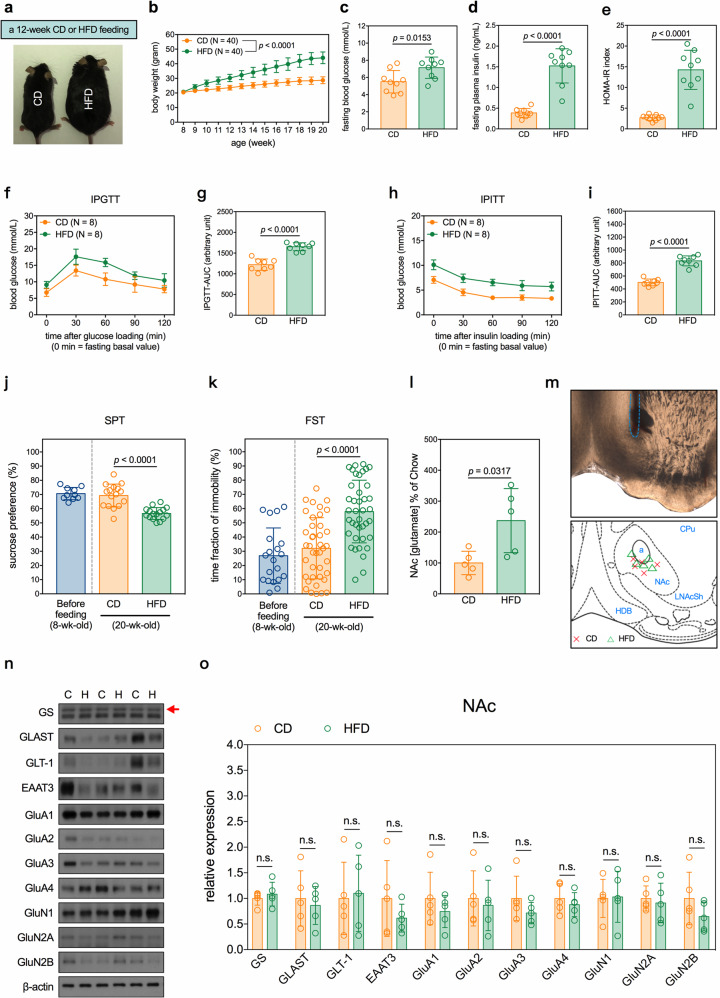
HFD induces MetD and depression-like behavior and enhances NAc glutamatergic inputs in mice. Effects of a 12-week HFD on parameters of interests. **a** Representative photograph of physical appearances of mice after the feedings. **b** Body weight of mice during the feedings. *n* = 40 mice per group. **c**–**e** Measurements of **e** fasting blood glucose levels, **d** fasting plasma insulin levels, and **e** HOMA-IR index in mice. *n* = 9 mice per group. Results of IPGTT. **f** Blood glucose levels during IPGTT in mice. **g** Analysis of area under the curve (AUC) of IPGTT results. *n* = 8 mice per group. Results of IPITT. **h** Blood glucose levels during IPITT in mice. **i** analysis of AUC of IPITT results. *n* = 8 mice per group. **j** Results of sucrose preference in SPT. *n* = 10 cages of mice in 8-wk-old group; *n* = 16 cages of mice in 20-wk-old CD and HFD groups. **k** Results of exhibition of immobility in FST. *n* = 20 mice in 8-wk-old group; *n* = 40 mice in 20-wk-old CD and HFD groups. Measurement of extracellular glutamate concentrations in the NAc of mice. **l** Quantitative results. *n* = 5 mice per group. **m** Locations of biosensor tips in real-time measurement of extracellular level of glutamate. Blue dash line indicates the inserting track in the upper panel. a: anterior commissure; CPu: caudate putamen; HDB: horizontal diagonal band of Broca; LNAcSh: NAc lateral shell. Measurements of expression of glutamatergic transmission-related molecules in the NAc of mice. **n** Representative micrographs of Western blots. The red arrow indicates the accurate molecular weight of GS. **o** Quantitative results. *n* = 5 samples per group. Each sample contained NAc protein lysates from 2 mice in equal amount of proteins. All data are expressed as mean ± SD. n.s., not significant. See also Supplementary Tables S1 and S2 for details of animal usage and statistical test results.

To delineate the mechanism underlying MetD-related depressive syndrome, we fed mice with a HFD for 12 weeks to induce MetD. Compared with mice fed with a CD, HFD mice had a significantly higher body weight gain (Fig. 1a, b), fasting blood glucose level (Fig. 1c), fasting plasma insulin level (Fig. 1d), and homeostatic model assessment-IR index (Fig. 1e) with impaired glucose [intraperitoneal glucose tolerance test, Fig. 1f, g] and insulin [intraperitoneal insulin tolerance test, Fig. 1h, i] tolerance. HFD mice also exhibited depression-like behavior as evidenced by their lower consumption of sucrose solution in the SPT (Fig. 1j), and higher immobilization time in the FST (Fig. 1k) than CD mice. The behavioral results (SPT and FST) of CD mice (20-week-old) were comparable to those of another group of 8-week-old mice (Fig. 1j, k), suggesting that age did not affect performance of CD mice in these two tests. Moreover, the exhibition of depression-like behaviors in the HFD mice could be alleviated by 4 weeks of treatment (20 mg/kg/day, i.p.) with fluoxetine, one of the most frequently prescribed antidepressants (Supplementary Fig. S1). Using real-time recording biosensors, we found that the HFD mice had increased levels of extracellular glutamate in the NAc (Fig. 1l, m), which is associated with the depressive phenotype onset [26, 32]. However, HFD did not affect the expressions of glutamine synthetase (GS) or glutamate transporters, including GLAST, GLT-1, and excitatory amino acid transporter (EAAT) 3 in the NAc (Fig. 1n, o). GLAST and GLT-1 are termed EAAT1 and EAAT2, respectively, in humans. The levels of subunits of ionotropic glutamate receptors (i.e., GluA1-4, GluN1, GluN2A, and GluN2B) in the NAc were also not affected by HFD (Fig. 1n, o). We then investigated whether increased glutamate concentrations in the NAc of HFD mice were derived from hyperactivated glutamatergic inputs.

### HFD induces hyperactivation of the vHPC→NAc glutamatergic projecting neurons in mice

**Fig. 2:**
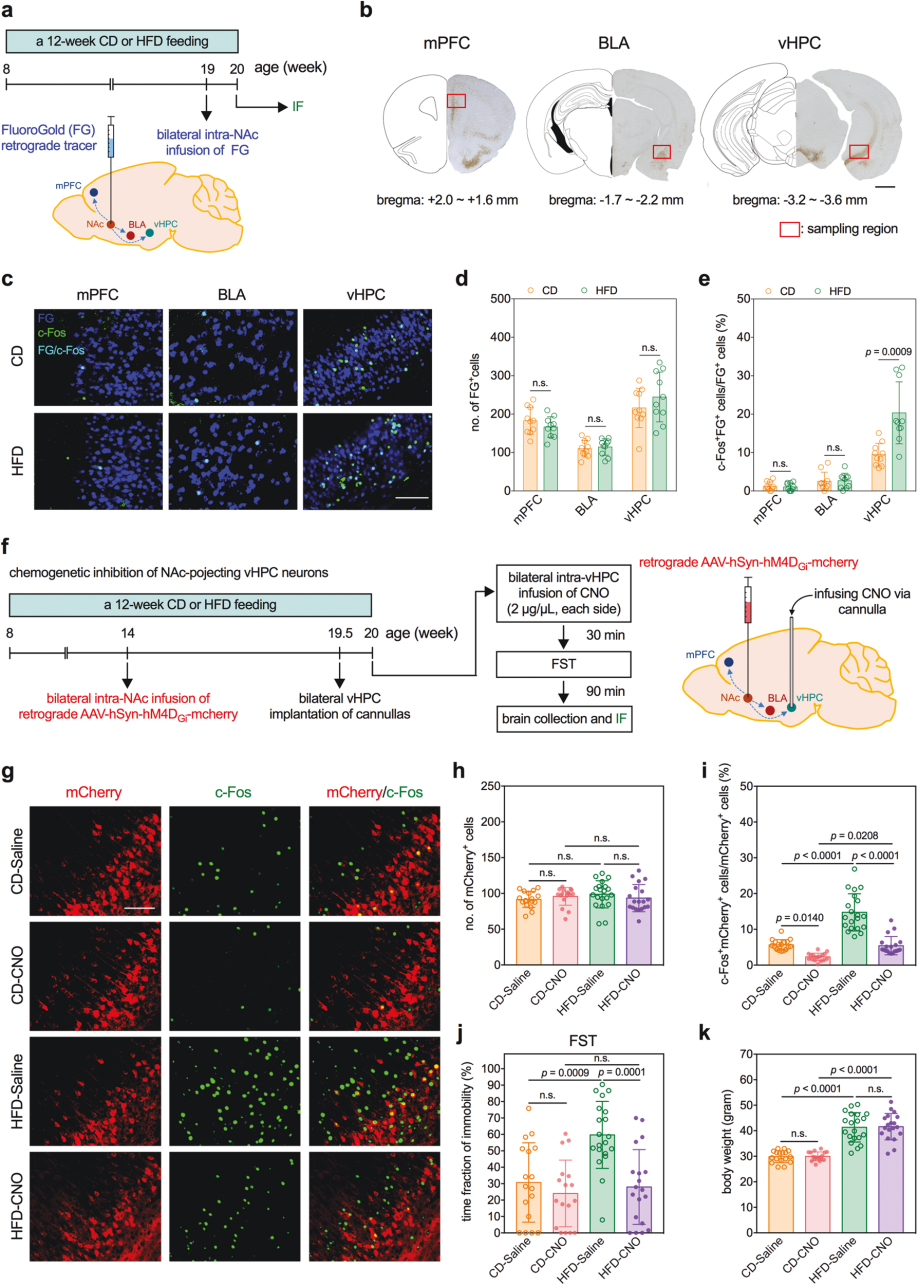
HFD induces hyperactivation of the vHPC→NAc projecting neurons, which contributes to the development of HFD-induced depressive phenotype in mice. **a** Experimental timeline and schematic of intra-NAc infusion of retrograde tracer, FG. **b** Representative images of immunohistochemistry of FG in the mPFC, BLA, and vHPC of mice that received intra-NAc infusion of FG. Red boxes indicate the sampled areas for cell counting in this study. Stereotaxic coordinates of each brain region are given beneath each region. Scale bar, 1 mm. Effects of a 12-week HFD on c-Fos expression in the FG-labeled NAc-projecting neurons in the mPFC, BLA, and vHPC of mice. **c** Representative fluorescence images of c-Fos-immunoreactive (green) and FG-labeled (blue) cells. Scale bar, 100 µm. **d** Number of FG-labeled cells. **e** Percentage of FG-labeled cells also expressing c-Fos. *n* = 10 mice per group. **f** Experimental timeline and schematic of chemogenetic inhibition of the vHPC→NAc glutamatergic neurons in mice. Effects of chemogenetic inhibition of the vHPC→NAc neurons on exhibition of depression-like behaviors in mice. **g** Representative fluorescence images of c-Fos-immunoreactive cells (green) and retrogradely labeled mCherry-expressing NAc-projecting neurons in the vHPC (red). Scale bar, 100 µm. **h** Number of mCherry-labeled NAc-projecting neurons in the vHPC. **i** Percentage of mCherry-labeled neurons also expressing c-Fos. **j** Results of FST. **k** Body weight of mice before FST. *n* = 17 mice in CD-Saline group; *n* = 17 mice in CD-CNO group; *n* = 20 mice in HFD-Saline group; *n* = 19 in HFD-CNO group. All data are expressed as mean ± SD. n.s. not significant. See also Supplementary Tables S1 and S2 for details of animal usage and statistical test results.

The NAc receives glutamatergic afferents primarily from the mPFC, BLA, and vHPC, which differentially regulate the exhibition of depression-like behavior [32–34]. To label the NAc-projecting neurons, a retrograde tracer, FG, was bilaterally infused into the NAc of mice (Fig. 2a). Our study revealed that an infusion of 0.03 µL of FG into the NAc resulted in a locally confined diffusion (Supplementary Fig. S2a) and successfully labeled cells in multiple brain regions that project to the NAc 1 week later. The FG-positive (FG^+^) cells were evident not only in the mPFC, BLA, and vHPC, where they almost exclusively expressed glutaminase, a glutamatergic neuron-specific marker, but also in the mediodorsal nucleus of the thalamus, pyramidal layer of the piriform area, and VTA (Supplementary Fig. S2b–d). In the sampled areas of mPFC, BLA, and vHPC (Fig. 2b), the number of FG^+^ cells was comparable between the HFD and CD groups (Fig. 2c, d). Among the FG^+^ cells, the ratios of cells stained positive for c-Fos, a marker for neuronal activation, were increased in the vHPC, but not the mPFC or BLA, of HFD mice (Fig. 2c, e). These findings suggest that the long-term consumption of HFD could induce the hyperactivation of the vHPC→NAc glutamatergic transmission.

### Hyperactivation of the vHPC→NAc glutamatergic projection contributes to the development of HFD-induced depressive phenotype in mice

To test whether the hyperactive vHPC→NAc glutamatergic projection is involved in the HFD-induced depressive phenotype, we employed chemogenetic inhibition to silence the vHPC→NAc glutamatergic neurons. The rAAVs expressed mCherry reporter and hM4D_Gi_ were bilaterally infused into the NAc to target the NAc-projecting neurons 6 weeks before ending HFD (Fig. 2f). The rAAV-infected NAc-projecting neurons were identified in the mPFC, BLA, vHPC, and piriform area (Supplementary Fig. S3a, b). The infected NAc-projecting neurons in the mPFC, BLA, and vHPC were also exclusively glutaminase-expressing glutamatergic neurons (Supplementary Fig. S3ac). Neither HFD nor bilateral intra-vHPC infusion of CNO affected the number of mCherry^+^ glutamatergic neurons in the vHPC (Fig. 2g, h and Supplementary Fig. S4a). However, bilateral intra-vHPC infusion of CNO significantly decreased the ratios of c-Fos^+^ cells in the vHPC mCherry^+^ cells in HFD mice (Fig. 2g, i and Supplementary Fig. S4a, HFD-saline vs. HFD-CNO) and decreased their immobility during the FST (Fig. 2j). The short half-life of the CNO (~2 h) [35] precludes the execution of the 24-h SPT. The infusion of CNO to the vHPC does not affect the numbers of mCherry^+^ cells or ratios of c-Fos^+^mCherry^+^/mCherry^+^ cells in the mPFC (Supplementary Fig. S4b–d) or BLA (Supplementary Fig. S4e–g) of HFD mice. Moreover, CNO did not affect the number of vHPC mCherry^+^ cells (Supplementary Fig. S5a, b), ratio of vHPC c-Fos^+^mCherry^+^/mCherry^+^ cells (Supplementary Fig. S5a, c), or depression-like behavior (Supplementary Fig. S5d) in HFD mice that were infected with rAAV expressing only mCherry (without hM4D_Gi_). These results indicated that chemogenetic inhibition-induced antidepressant-like effects were not derived from CNO alone. Finally, CNO treatment did not alter the body weights in either CD or HFD mice infected with rAAV expressed with or without hM4D_Gi_ (Fig. 2k and Supplementary Fig. S5e). Altogether, these results demonstrated that hyperactive vHPC→NAc glutamatergic projection contributes to the HFD-induced depressive phenotype in mice.

### HFD downregulates glutamate transporters, GLAST and GLT-1, in the vHPC

**Fig. 3:**
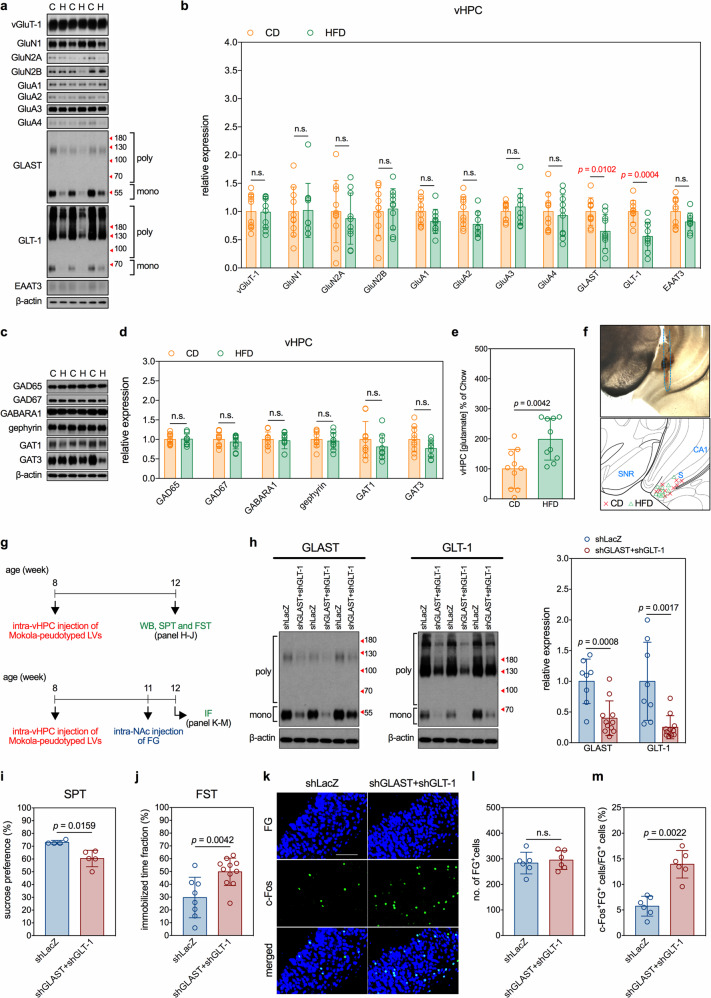
HFD downregulates glutamate transporters, GLAST and GLT-1, in the vHPC of mice and knockdown of the vHPC GLAST and GLT-1 reproduces HFD-related circuit and behavioral impairments in naïve mice. Effects of a 12-week HFD on parameters of interests. **a** and **b** Measurements of expression of glutamatergic transmission-related molecules in the vHPC of mice. **a** Representative micrographs of Western blots. **b** Quantitative results. *n* = 10 mice per group. Measurements of expression of GABAergic transmission-related molecules in the vHPC of mice. **c** Representative micrographs of Western blots. **d** Quantitative results. *n* = 10 mice per group. Measurement of extracellular glutamate concentrations in the vHPC of mice. **e** Quantitative results. *n* = 10 mice per group. **f** Locations of biosensor tips in real-time measurement of extracellular level of glutamate. Blue dash line indicates the inserting track in the upper panel. S: subiculum; CA1: cornu ammonis 1; SNR: substantia nigra pars reticulata. **g** Experimental timelines of studies determining effects of lentiviral knockdown of vHPC GLAST and GLT-1 on activity of vHPC→NAc glutamatergic transmission and depression-like behaviors in naïve mice. **h** Efficacy of lentiviral knockdown of vHPC GLAST and GLT-1. Left panels show representative micrographs of Western blots. Right panel shows quantitative results. *n* = 8 mice in shLacZ group; *n* = 11 mice in shGLAST+shGLT-1 group. **i** Results of SPT. *n* = 4 cages of mice in shLacZ group; *n* = 5 cages of mice in shGLAST+shGLT-1 group. **j** Results of FST. *n* = 8 mice in shLacZ group; *n* = 11 mice in shGLAST+shGLT-1 group. Measurements of c-Fos expression in the FG-labeled vHPC→NAc glutamatergic neurons in naïve mice. **k** Representative fluorescence images of c-Fos-immunoreactive cells (green) and FG-labeled NAc-projecting cells (blue) in the vHPC. Scale bar, 100 µm. **l** Number of FG-labeled cells. **m** Percentage of FG-labeled cells also expressing c-Fos. *n* = 6 mice per group. All data are expressed as mean ± SD. n.s. not significant. See also Supplementary Tables S1 and S2 for details of animal usage and statistical test results.

Hyperactive glutamatergic transmission may result from imbalanced glutamatergic and GABAergic transmissions that have been identified in patients with depression [36]. To explore possible mechanisms causing the hyperactive vHPC→NAc transmission, we determined the expression levels of a series of key molecules mediating the glutamatergic and GABAergic neurotransmissions in the vHPC of CD and HFD mice. The glutamatergic presynaptic protein, vesicular glutamate transporter-1 (vGluT-1), the subunits of postsynaptic ionotropic glutamate receptors, including GluA1-4, GluN1, GluN2A, and GluN2B, and the glutamate transporters, GLAST, GLT-1, and EAAT3, were selected to evaluate the glutamatergic transmission, whereas, the two isoforms of glutamic acid decarboxylase (GAD) responsible for GABA synthesis, GAD65 and GAD67, the postsynaptic α-1 subunit of ionotropic GABA_A_ receptor (GABARA1) and GABA_A_ receptor cluster, gephyrin, and the GABA transporters, GAT1 and GAT3, were selected to evaluate the GABAergic transmission. Results showed that, among these selected key molecules, only the levels of GLAST and GLT-1 in the vHPC were significantly altered (downregulated) by long-term HFD (Fig. 3a–d).

GLAST and GLT-1, mainly expressed on astroglia, are the predominant glutamate transporters accounting for nearly all synaptic clearance of glutamate in the cerebrum [37, 38]. In line with the inverted relationship between levels of glial glutamate transporters and neuronal activity [39–41], we detected an increase in the extracellular glutamate concentrations in the vHPC of HFD mice (Fig. 3e, f). HFD-related downregulation of GLAST and GLT-1 was not evident in the mPFC or BLA (Supplementary Fig. S6).

### HFD-induced decreases in vHPC GLAST and GLT-1 lead to hyperactivation in the vHPC→NAc glutamatergic transmission and depression-like behavior

To test the causal relationship between the downregulation of GLAST and GLT-1 in the vHPC and depressive phenotypes, LVs expressing shGLAST, shGLT-1, and LacZ were pseudotyped with the glycoprotein of Mokola virus to enhance the tropism to astrocytes (Supplementary Fig. S7a, b) and injected in naïve mice. Four weeks after the bilateral infusion of LVs expressing shGLAST+shGLT-1 into the vHPC of 8-week-old naïve mice (Fig. 3g), the levels of GLAST and GLT-1 in the vHPC were reduced (Fig. 3h). These mice also expressed depression-like behavior, including decreased sucrose consumption in the SPT (Fig. 3i) and increased immobilization time in the FST (Fig. 3j). Retrograde FG tracing showed that the knockdown of GLAST and GLT-1 did not change the numbers of FG^+^ cells (Fig. 3k, l) but increased the ratios of c-Fos^+^FG^+^ cells in the vHPC (Fig. 3k, m). These findings indicate that decreased levels of GLAST and GLT-1 in the vHPC induce depression-like behavior in naïve mice.

**Fig. 4:**
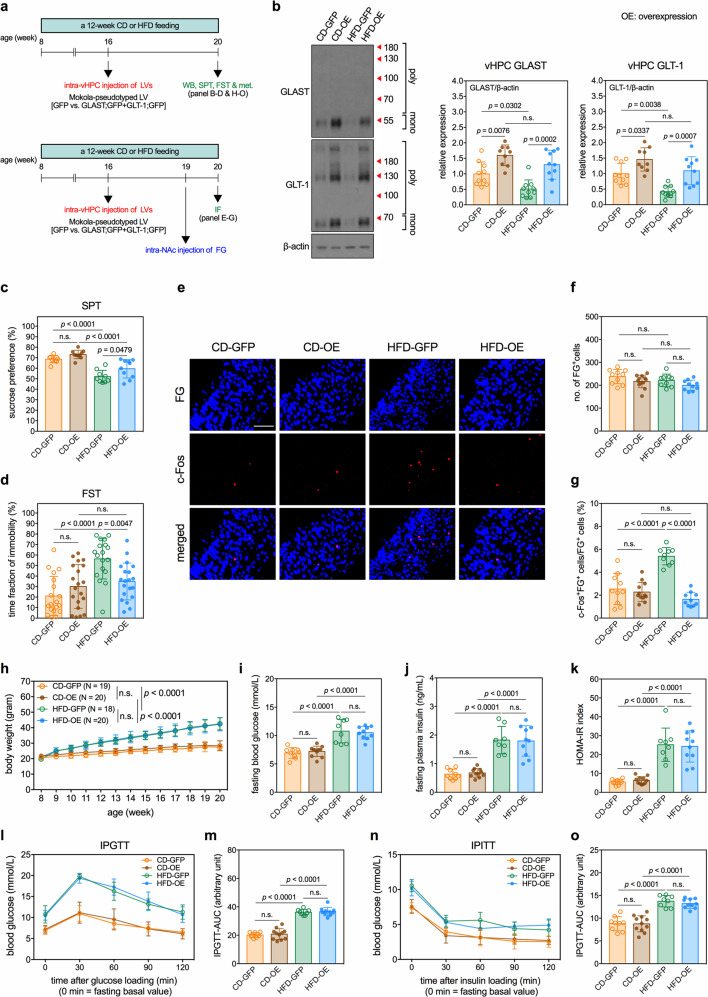
Restoring the vHPC GLAST and GLT-1 reverses HFD-induced hyperactivation in the vHPC→NAc glutamatergic transmission and depression-like behavior. **a** Experimental timelines of studies determining effects of lentiviral overexpression of vHPC GLAST and GLT-1 on activity of vHPC→NAc glutamatergic transmission and depression-like behaviors in CD and HFD mice. **b** Efficacy of lentiviral overexpression of vHPC GLAST and GLT-1. Left panels show representative micrographs of Western blots. Right panels shows quantitative results. *n* = 10 mice in CD-GFP group; *n* = 9 mice in CD-OE group (OE: overexpression); *n* = 10 mice in HFD-GFP group; *n* = 10 mice in HFD-OE group. **c** Results of SPT. *n* = 9 cages of mice in CD-GFP group; *n* = 10 cages of mice in CD-OE group; *n* = 9 cages of mice in HFD-GFP group; *n* = 10 cages of mice in HFD-OE group. **d** Results of FST. *n* = 19 mice in CD-GFP group; *n* = 20 mice in CD-OE group; *n* = 18 mice in HFD-GFP group; *n* = 20 mice in HFD-OE group. Measurements of c-Fos expression in the FG-labeled NAc-projecting neurons in the vHPC of mice. **e** Representative fluorescence images of c-Fos-immunoreactive cells (green) and FG-labeled NAc-projecting cells (blue) in the vHPC. Scale bar, 100 µm. **f** Number of FG-labeled cells. **g** Percentage of FG-labeled cells also expressing c-Fos. *n* = 10 mice in CD-GFP group; *n* = 12 mice in CD-OE group; *n* = 9 mice in HFD-GFP group; *n* = 10 mice in HFD-OE group. **h** Body weight of mice during the experimental period. *n* = 19 mice in CD-GFP group; *n* = 20 mice in CD-OE group; *n* = 18 mice in HFD-GFP group; *n* = 20 mice in HFD-OE group. Measurements of **i** fasting blood glucose levels, **j** fasting plasma insulin levels, and **k** HOMA-IR index in mice. *n* = 9 mice in CD-GFP group; *n* = 11 mice in CD-OE group; *n* = 8 mice in HFD-GFP group; *n* = 10 mice in HFD-OE group. Results of IPGTT. **l** Blood glucose levels during IPGTT in mice. **m** Analysis of AUC of IPGTT results. *n* = 9 mice in CD-GFP group; *n* = 11 mice in CD-OE group; *n* = 8 mice in HFD-GFP group; *n* = 10 mice in HFD-OE group. Results of IPITT. **n** Blood glucose levels during IPITT in mice. **o** Analysis of AUC of IPITT results. *n* = 9 mice in CD-GFP group; *n* = 11 mice in CD-OE group; *n* = 8 mice in HFD-GFP group; *n* = 10 mice in HFD-OE group. All data are expressed as mean ± SD. n.s., not significant. See also Supplementary Tables S1 and S2 for details of animal usage and statistical test results.

Next, we tested whether GLAST and GLT-1 level restorations in the vHPC inhibited depression-like behavior in HFD mice. LVs expressing GLAST, GLT-1, and green fluorescent protein (GFP) reporter were generated (Supplementary Fig. S7c, d) and bilaterally infused into the vHPC of mice 4 weeks before the end of the 12-week HFD feeding period (Fig. 4a). Mice that received infusions of LVs expressing GLAST and GLT-1 expressed higher levels of GLAST and GLT-1 in the vHPC than their respective GFP control mice (Fig. 4b). Restoring the expression levels of GLAST and GLT-1 in the vHPC decreased the depression-like behavior (Fig. 4c, d) and the activities of vHPC→NAc transmission (Fig. 4e–g) in HFD mice. Overexpressing GLAST and GLT-1 in the vHPC of CD mice did not lead to depression-like behavior in them (Fig. 4c, d) or the activities of vHPC→NAc transmission (Fig. 4e–g). Furthermore, the overexpression of GLAST and GLT-1 in the vHPC did not affect body weight or insulin resistance in CD or HFD mice (Fig. 4h–o). These results demonstrate that downregulating GLAST and GLT-1 in the vHPC causes hyperactivation of the vHPC-NAc glutamatergic transmission, which leads to the depressive phenotype in HFD mice.

### Riluzole normalizes HFD-induced hyperactivation of glutamatergic transmissions and depression-like behavior

**Fig. 5:**
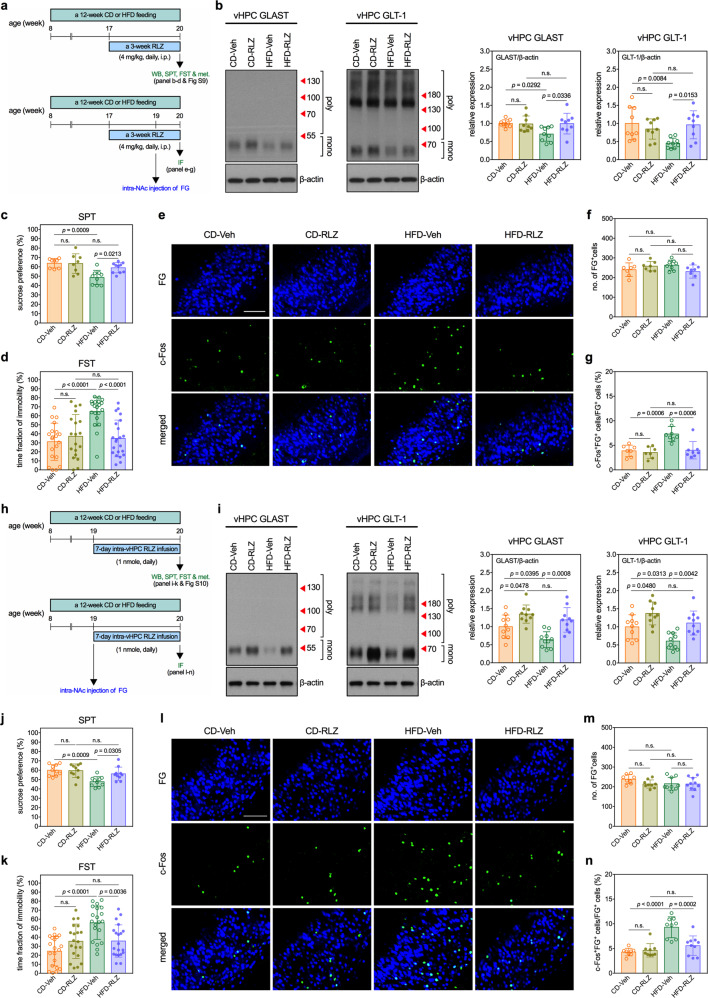
Riluzole corrects HFD-induced hyperactivation of glutamatergic transmissions and depression-like behaviors. **a** Experimental timelines of studies determining effects of a 3-week systemic (intraperitoneal injection) riluzole (RLZ) treatment on expressions of GLAST and GLT-1 in the vHPC, activity of vHPC→NAc glutamatergic transmission, and depression-like behaviors in CD and HFD mice. **b** Measurements of expressions of GLAST and GLT-1 in the vHPC of mice. Left panels show the representative micrographs of Western blots. Right panel shows quantitative results. *n* = 9 mice per group. **c** Results of SPT. *n* = 8 cages of mice in CD-Veh group (Veh: vehicle control); *n* = 8 cages of mice in CD-RLZ group; *n* = 9 cages of mice in HFD-Veh group; *n* = 10 cages of mice in HFD-RLZ group. **d** Results of FST. *n* = 20 mice in CD-Veh group; *n* = 19 mice in CD-RLZ group; *n* = 20 mice in HFD-Veh group; *n* = 22 mice in HFD-RLZ group. Measurements of c-Fos expression in the FG-labeled vHPC→NAc glutamatergic neurons in mice. **e** Representative fluorescence images of c-Fos-immunoreactive cells (green) and FG-labeled NAc-projecting cells (blue) in the vHPC. Scale bar, 100 µm. **f** Number of FG-labeled cells. **g** Percentage of FG-labeled cells also expressing c-Fos. *n* = 7 mice in CD-Veh group; *n* = 7 mice in CD-RLZ group; *n* = 8 mice in HFD-Veh group; *n* = 8 mice in HFD-RLZ group. **h** Experimental timelines of studies determining effects of a 7-day central (intra-vHPC infusion) RLZ treatment on expressions of GLAST and GLT-1 in the vHPC, activity of vHPC→NAc glutamatergic transmission, and depression-like behaviors in CD and HFD mice. **i** Measurements of expressions of GLAST and GLT-1 in the vHPC of mice. Left panels show the representative micrographs of Western blots. Right panel shows quantitative results. *n* = 10 mice per group. **j** Results of SPT. *n* = 10 cages per group. **k** Results of FST. *n* = 20 mice per group. Measurements of c-Fos expression in the FG-labeled vHPC→NAc glutamatergic neurons in mice. **l** Representative fluorescence images of c-Fos-immunoreactive cells (green) and FG-labeled NAc-projecting cells (blue) in the vHPC. Scale bar, 100 µm. **m** Number of FG-labeled cells. **n** Percentage of FG-labeled cells also expressing c-Fos. *n* = 8 mice in CD-Veh group; *n* = 9 mice in CD-RLZ group; *n* = 9 mice in HFD-Veh group; *n* = 10 mice in HFD-RLZ group. All data are expressed as mean ± SD. n.s., not significant. See also Supplementary Tables S1 and S2 for details of animal usage and statistical test results.

For potentially treating MetD-related depressive syndrome, we screened for compounds that improve the functional capacity of glutamate clearance. Daily systemic injection of RLZ (4 mg/kg, i.p.) for 3 weeks to the HFD mice at the last phase of the 12-week feeding period (Fig. 5a) significantly increased levels of GLAST and GLT-1 in the vHPC (Fig. 5b). Moreover, RLZ treatment ameliorated the depression-like behavior in the SPT (Fig. 5c) and FST (Fig. 5d) and repressed the activity of the FG-labeled vHPC→NAc glutamatergic neurons (Fig. 5e–g) in HFD mice.

RLZ is known to affect locomotor networks of the spinal cord and peripheral nervous system [42–44]. To prevent potential confounding peripheral effects on mobility, we directly infused 1 nmol of RLZ into both vHPCs of mice each day for the last 7 days of HFD feeding period (Fig. 5h). Our initial experiments showed that this dosing schedule was effective at upregulating GLAST and GLT-1 in the vHPC (Supplementary Fig. S8). Similar to systemic RLZ treatment, intra-vHPC infusion of RLZ increased the levels of GLAST and GLT-1 in the vHPC of both CD and HFD mice (Fig. 5i), alleviated HFD-induced depression-like behaviors in the SPT (Fig. 5j) and FST (Fig. 5k), and suppressed HFD-induced hyperactivity in vHPC→NAc glutamatergic transmission (Fig. 5l–n). Moreover, RLZ administered through either route had no effects on body weight or insulin resistance in CD or HFD mice (Supplementary Figs. S9 and 10). Thus, the therapeutic benefits of RLZ in combatting HFD-induced depression-like phenotypes were not likely to result from effects on metabolism dysregulation; rather, the effects probably resulted from the attenuation of hyperactive vHPC→NAc glutamatergic transmission.

## Discussion

Depressive syndrome is frequently accompanied by MetD with an unclear mechanism. Herein, we demonstrated that astroglia-associated perturbations in the vHPC→NAc glutamatergic circuit contributed to the onset of depression-like phenotype in a mouse model of diet-induced obesity/MetD. Disturbed glutamatergic transmission has been linked to depressive disorders [45]. These include altered glutamate levels and the expressions of N-methyl-D-aspartate receptor subunits in patients with depression [46]. Results derived from various depression animal models also support a vital role of corticolimbic glutamatergic transmission in the pathogenesis of depressive disorders, and controlling this neural circuit represents a common pathway for the therapeutic action of antidepressants [47]. Furthermore, impaired glutamate clearance resulting from dysfunctions of glial glutamate transporters has been implicated in depression [48–50]. Reduced levels of EAAT1 and 2 were found in suicidal patients with major depression disorders [51]. GLT-1 levels are negatively correlated with the exhibition of helpless behavior in stressed rats [52]. Pharmacological blockade of central GLT-1 induces anhedonia in rats [53–55]. These findings imply that disturbed expressions of GLAST and GLT-1 and subsequent hyperactive glutamatergic transmission represent a seminal pathological pathway for MetD-related depressive syndrome.

The three major glutamatergic inputs to the NAc differentially regulate the manifestation of depressive phenotypes. Increases in vHPC→NAc glutamatergic neuronal activities by optogenetic induction trigger depression-related behavioral abnormalities in mice, while reduced activities in vHPC→NAc glutamatergic neurons are associated with resilience to CSDS-induced depression-like social avoidance behaviors [32]. Conversely, optogenetic activations of glutamatergic neurons projecting to the NAc from the mPFC and BLA alleviate depression-like behavior in mice susceptible to CSDS [32, 33]. The activation of the vHPC→NAc neurons increases the activity of VTA→NAc DA neurons [56, 57], a leading cause of susceptibility in the CSDS depression model [23, 24, 58]. Furthermore, long-term consumption of HFD induces maladaptation in the VTA→NAc DA reward circuit (i.e., increased levels of brain-derived neurotrophic factor and decreased expression ratios of DA receptors, D1R to D2R, in the NAc) [15]. These findings strongly suggest that the hyperactivation of the vHPC→NAc glutamatergic transmission determines the inception of MetD-related depressive syndrome. Furthermore, enhanced brain-derived neurotrophic factor signal pathway and imbalanced D1R/D2R signaling in the NAc are both evident in mice exposed to CSDS [59–61] and fed with HFD [15]. It appears that chronic stress and chronic consumption of an unhealthy diet share a common pathogenic pathway for the onset of depressive syndrome. Whether downregulation of GLAST and GLT-1 in astrocytes also play an initiation role in the onset of chronic stress-induced depression-like behaviors deserves future investigation.

Several antidepressants acting via increasing the synaptic availability of monoamines are widely used to treat depressive syndrome [62], although a number of patients show poor responses to these first-line pharmacological interventions [63]. With a huge diversity in etiology and neurotransmission systems involved in the pathogenesis of depressive mood disorder, it is crucial to understand the pathological mechanisms of depressive mood disorder induced by various factors and to identify potential drugs suitable for specific impaired components in different subtypes of depressive mood disorder. We highlighted the therapeutic potential of RLZ in MetD-related depressive syndrome by restoring the expressions of GLAST and GLT-1 and the activity of vHPC→NAc glutamatergic transmission. RLZ, the first approved drug for amyotrophic lateral sclerosis, enhances the activities and expressions of GLAST and GLT-1 in astrocytes [64–68]. The regulatory effect of RLZ on glutamatergic transmission have been exploited to treat patients with depressive syndrome [69–72]. However, a portion of patients with depressive syndrome do not respond to RLZ [73, 74], as is the case with most other antidepressants. It may therefore be of clinical interest to further investigate the effects of RLZ in patients with comorbid depression and MetD who also have impaired glial glutamate reuptake. It is worth noting that our 3-week systemic injection of RLZ caused a relatively high death rate (8/34, ≈ 24%) in CD mice, whereas none of the CD mice receiving 7-day intra-vHPC infusion of RLZ died. Moreover, none of the HFD mice that received either the peripheral injections or repeated intra-vHPC infusions of RLZ died. Thus, further investigations may be warranted to test whether chronic systemic RLZ treatment might compromise life-sustaining glutamatergic transmission.

The HFD-induced depression-like behaviors could be alleviated by a 4-week treatment of fluoxetine, a selective serotonin reuptake inhibitor (SSRI). However, fluoxetine is known to improve HFD-induced MetD outcomes, including overweight, hypertrophy of adipose tissues, dyslipidemia, and systemic insulin resistance [9]. These findings are in accordance with previous clinical observations that SSRIs show a benefit for blood glucose control in adults with major depressive disorder comorbid with type 2 diabetes [10, 11]. The pleiotropic effects of fluoxetine make it difficult to discern whether the drug acted in our experiment by enhancing the central serotonin transmission, improving peripheral metabolic dysfunction, or a combination of both effects. To focus on the central nervous system regulation of depressive phenotypes, we directly targeted glutamatergic afferents to the NAc.

Among the three major glutamatergic inputs to the NAc, downregulation of glial GLAST and GLT-1 were only observed in the vHPC, but not in the mPFC or BLA of HFD mice, indicating a region-specific vulnerability of astrocytes to the HFD. Astrocytes have diverse functions and phenotypes [75–77] and express a broad range of molecular markers, such as GFAP, S100β, connexin-43, and aldehyde dehydrogenase-1 family member L1 [75, 78]. Interestingly, GFAP expression in the mouse brain is unevenly distributed, which has been associated with regional proliferation of the embryonic neuroepithelium [79, 80]. Long-term HFD feeding alters the morphology of GFAP^+^ astrocytes in the hippocampus [81], while mutations in GFAP disrupt the lipid biosynthesis in astrocytes [82]. In the mouse brain, the hippocampus is one of the few exceptions expressing remarkably abundant and regular staining patterns of GFAP [79, 80]. The unique feature of GFAP^+^ astrocytes in lipid metabolism and high GFAP-expression profile in the hippocampus may render astrocytes of this region more sensitive to HFD-related insults than other low-GFAP-expressing regions.

The time of immobility is considered an indicator in the FST. Because the mobility of mice may be affected by body weight, we paid attention to this potential confounder. In the experiments of restoring expressions of GLAST and GLT-1 in the vHPC, both genetic and pharmacological approaches could block HFD-induced increases in immobility time during the FST but did not influence HFD-related body weight gain. Likewise, increased FST immobility in HFD mice could be rescued by chemogenetic inhibition of the vHPC→NAc glutamatergic circuit without affecting their body weight. These results indicate that the HFD-induced increases in immobility time during the FST was primarily attributed to hyperactive vHPC→NAc glutamatergic circuit caused by downregulation of GLAST and GLT-1, rather than overweight.

There are several limitations to this study. First, the best behavioral tests to measure depressive phenotypes in rodent models are currently under debate. We used SPT and FST as the major behavioral tests in this study. While the FST was originally developed to study the efficacy of antidepressants, it has been recently challenged as being a good test for assessing depression induction [83]. Second, we observed that both genetic overexpression and intra-vHPC infusion of RLZ could upregulate GLAST and GLT-1 levels in the vHPC, but these manipulations did not affect the activity of the vHPC→NAc glutamatergic transmission or the results of the SPT and FST in the CD control mice. This lack of effects may be due to the fact that CD mice exhibit highly efficient glutamate clearance and ceiling/floor effects of sucrose consumption in the SPT/immobility time in the FST. Thus, it is difficult to use genetic or pharmacological approaches to enhance glutamate clearance or to push the behavioral readouts past the baseline ceiling/floor levels. Third, it has been shown that sex differences exist in clinical depression [84], and preclinical animal models also exhibit sexual dimorphisms in depression-like behaviors [85, 86]. Moreover, GLAST and GLT-1 levels are known to be positively regulated by estrogen [87–92]. To avoid the potential confounding effects of sex, only male mice were used in this study. Thus, it is possible that our findings in male mice may not be completely recapitulated in female mice. Finally, we used an intra-cranial biosensor to monitor the levels of extracellular glutamate. However, some experiments (i.e., lentiviral knockdown and overexpression and intra-vHPC infusion of RLZ) require multiple infusions into the vHPC, which may damage brain tissues and influence local glutamate concentrations. Therefore, we did not measure extracellular glutamate concentrations in these cases. These limitations should be taken into consideration when interpreting our findings.

In conclusion, long-term consumption of HFD downregulated the expressions of glial glutamate transporters, GLAST and GLT-1, in the vHPC, which led to ineffective glutamate clearance and hyperactive vHPC→NAc glutamatergic transmission, leading to depression-like behavior. RLZ, given systematically, restored expressions of GLAST and GLT-1 in the vHPC, normalizing the activity of vHPC→NAc glutamatergic transmission and decreasing depression-like behavior. This study not only provides the circuit and molecular mechanisms underlying MetD-related depressive syndrome, but also advises a therapeutic choice for this specific type of depressive mood disorder, highlighting the translational potential of this study.

## Supplementary information

Supplementary 1

Supplementary Figure S1

Supplementary Figure S2

Supplementary Figure S3

Supplementary Figure S4

Supplementary Figure S5

Supplementary Figure S6

Supplementary Figure S7

Supplementary Figure S8

Supplementary Figure S9

Supplementary Figure S10

Supplementary Table S1

Supplementary Table S2
