# Supplementary material for: High-fat diet induces depression-like phenotype via astrocyte-mediated hyperactivation of ventral hippocampal glutamatergic afferents to the nucleus accumbens
Source: Mol Psychiatry. 2022 Sep 30;27(11):4372–84. doi: 10.1038/s41380-022-01787-1 (PMC9734059; doi:10.1038/s41380-022-01787-1)
Supplement: Supplementary file 1 — Supplementary 1 [file 41380_2022_1787_MOESM1_ESM.docx]

| **Supplementary 1**  **Supplemental Methods and Other Relevant Information** |
| --- |

**High-fat diet induces depression via astrocyte-mediated hyperactivation of the ventral hippocampal glutamatergic afferents to the nucleus accumbens**

Sheng-Feng Tsai,^1,2^ Pei-Ling Hsu,^1,3,4^ Yun-Wen Chen,^1,5^ Mohammad Shahadat Hossain,^2,6^ Pei-Chun Chen,^1,3^ Shun-Fen Tzeng,^1,7^ Po-See Chen,^8,9^ and Yu-Min Kuo^1,2,^*

^1^Institute of Basic Medical Sciences, College of Medicine, National Cheng Kung University, Tainan, Taiwan

^2^Department of Cell Biology and Anatomy, College of Medicine, National Cheng Kung University, Tainan, Taiwan

^3^Department of Physiology, College of Medicine, National Cheng Kung University, Tainan, Taiwan

^4^Department of Anatomy, School of Medicine, College of Medicine, Kaohsiung Medical University, Kaohsiung, Taiwan

^5^Department of Pharmacology, College of Medicine, National Cheng Kung University, Tainan, Taiwan

^6^Interdisciplinary Neuroscience, Taiwan International Graduate Program, Academia Sinica, National Cheng Kung University, Tainan, Taiwan

^7^Department of Life Sciences, College of Bioscience and Biotechnology, National Cheng Kung University, Tainan, Taiwan

^8^Department of Psychiatry, College of Medicine, National Cheng Kung University, Tainan, Taiwan

^9^Addiction Research Center, National Cheng Kung University Hospital, National Cheng Kung University, Tainan, Taiwan

****Correspondence***

Yu-Min Kuo, Ph.D.

Department of Cell Biology and Anatomy

National Cheng Kung University Medical College

1 Ta Hsueh Road, Tainan, 70101

Taiwan

E-mail address: kuoym@mail.ncku.edu.tw

Tel.: +886-6-2353535 ext. 5294; fax: +886-6-2093007.

| **Contents of Supplementary 1**   1. Supplemental Methods 2. Legends for Supplementary Figures 3. Supplementary Table S3 4. Data and Resource Availability |
| --- |

**1. Supplemental Methods**

**EXPERIMENTAL MODEL AND SUBJECT DETAILS**

**Animals**

All animal experiments were approved by the National Cheng Kung University Institutional Animal Care and Use Committee (IACUC approval number: 104243, 106057, and 109139) and were in accordance with the National Institutes of Health Guide for the Care and Use of Laboratory Animals. Animals were obtained from and maintained in National Cheng Kung University Laboratory Animal Center (NCKULAC, Tainan, Taiwan) accredited by AAALAC. Mice were housed in ventilated cages (4-5 mice per cage) in a humidity- (55 ± 10%) and temperature-controlled (24 ± 1°C) specific-pathogen-free breeding unit of NCKULAC on a 12-h light/12-h dark cycle (light on at 7 AM). They had free access to water and diet unless food was withdrawn for fasting. The housing environment and animal health were monitored by qualified staff and veterinaries.

The computer-based randomization [random number generator in Microsoft Excel software (Microsoft Corporation, Redmond, WA, USA)] was used to assign animal groups. The animal groups were blinded to the investigators during the FST studies; no other studies were blinded to the investigators.

**HFD-induced obesity model**

Male C57BL/6N mice (8-week-old) were randomly assigned to CD and HFD groups with computer-based randomization [random number generator in Microsoft Excel software (Microsoft Corporation, Redmond, WA, USA)]. This method was used for animal grouping throughout the entire study. The CD group mice were fed with an autoclaved regular rodent diet (Cat# 5010, LabDiet, St. Louis, MO, USA), while the HFD group mice were fed with commercial HFD (60% energy from fat, Cat# 58Y1, TestDiet, St. Louis, MO, USA) for 12 weeks (from 8- to 20-week-old). Their food intakes were recorded daily while body weights were recorded weekly.

**Fasting blood glucose and plasma insulin levels**

After a 12-h fast, blood specimens were collected from mice by tail incision using heparinized capillary tubes (Cat# 563, Glaswarenfabrik Karl Hecht, Sondheim vor der Rhön, Bavaria, Germany). A small portion of the blood was used for detecting glucose levels with a glucose meter (Model: Accu-Chek Active^®^, Roche). The remaining blood was centrifuged at 3,000 × g for 10 min to collect the plasma. The plasma levels of insulin were measured using a commercial mouse insulin ELISA kit (Cat# 10-1247-01, Mercodia, Uppsala, Sweden).

**Calculation of HOMA-IR**

HOMA-IR index was calculated using the following formula: fasting glucose (mM) × fasting insulin (mU/L)/22.5.

**Glucose and insulin tolerance tests**

IPGTT and IPITT were performed in mice after they had fasted for 12 h. The mice were injected (i.p.) with 1.0 g/kg glucose in the IPGTT and 1.0 U/kg insulin in the IPITT. Blood specimens were collected by tail incision before and 30, 60, 90, and 120 min after the injections. The glucose levels of the collected blood were immediately detected with a glucose meter (Model: Accu-Chek Active^®^, Roche).

**SPT**

To avoid isolation that may affect the mood, we housed two to three mice in a cage during the test. Three days before the SPT, mice (housed 4–5/cage) were randomly divided into two cages. After a 3-day acclimation for the change of cagemate composition, mice were provided with two identical water bottles, one filled with distilled drinking water and the other with palatable 1% sucrose (Cat# S9378, Sigma-Aldrich, St. Louis, MO, USA) solution for 24 h. To minimize side preference, these two bottles were switched every 12 h. Consumptions of sucrose solution and water were determined by changes in fluid weight. Sucrose preference was defined as the ratio of the consumed weight of sucrose solution to the total consumed weight of sucrose solution and water during the 24 h. The result obtained from each cage was considered one data point.

**FST**

FST was conducted in a glass cylindrical tank (height: 30 cm, diameter: 20 cm) filled with temperature-controlled water (23–25°C) of a 15-cm height. Each mouse was placed in the water tank for 6 min; only the behavior of the last 4 min was analyzed by an experienced experimenter blinded to the animal grouping. The immobility time was presented as % of immobility time in 4 min.

**Fluoxetine treatment**

Fluoxetine (Cat# 1279804, Sigma-Aldrich) was prepared in normal saline and intraperitoneally injected into mice at dosage of 20 mg/kg/day for 4 weeks in the last phase of the HFD regimen. Vehicle control mice received saline injections of equal volume on the same schedule.

**Measurement of extracellular glutamate levels**

Glutamate biosensors (Cat# 7004, Pinnacle Technology, Lawrence, KS, USA) and a matched recording system (Cat# 8400-K1, Pinnacle Technology) were used to monitor the extracellular concentrations of glutamate in the nucleus accumbens (NAc) and ventral hippocampus (vHPC) of mice in real time. One week before ending the HFD, each mouse was implanted with a guiding cannula (Cat# 7034, Pinnacle Technology) either into the NAc [anteroposterior (AP): +1.0 mm; mediolateral (ML): +1.0 mm; dorsoventral (DV): -3.6 mm from the bregma] or the vHPC (AP: -3.2 mm; ML: +2.8 mm; DV: -3.8 mm from the bregma). The implanted cannula was tightly fixed on the mouse skull with dental adhesive resin cement (Model: Super-Bond, Sun Medical, Shiga, Japan). One week later, a glutamate biosensor (1 mm longer than the guiding cannula) was inserted into the cannula, and the mouse was allowed to move freely. Recording began after the readings stabilized and lasted for 30 min. Recordings pre- and post-calibration were carried out with the same digital signal amplifier that was used to validate the function of the biosensors and plot the standard curves for estimating the basal extracellular level of glutamate in the NAc and vHPC. Mice were euthanized after the recordings. Further, their brains were removed, and coronal sections were prepared using a vibratome (Model: DTK-1000; DOSAKA, Kyoto, Japan). The trace made by the insertion of a biosensor on the sections was used to mark the recording site.

**Western blot**

Mice were anesthetized with Zoletil 50 (75 mg/kg, i.p.; Virbac, Carros, France) and transcardially perfused with chilled phosphate-buffered saline (PBS, Cat# 10010023, Thermo Fisher Scientific). The brains were quickly dissected out, immediately immersed in liquid nitrogen, and stored at -80°C. The frozen tissues were homogenized with chilled T-PER lysis buffer (Cat# 78510, Thermo Fisher Scientific) containing protease inhibitors (Cat# 04693116001, Roche) and phosphatase inhibitors (Cat# 04906837001, Roche). The cell samples were lysed with chilled RIPA buffer (Cat# 89900, Thermo Fisher Scientific) containing the same protease and phosphatase inhibitors. The homogenates were centrifuged at 15,000 × g at 4°C for 15 min, and the supernatants were collected and subjected to protein estimation using a BCA kit (Cat# 23225, Thermo Fisher Scientific) and adjusted to the same concentration. The supernatants (10 μg of total protein) were mixed with a sample buffer (Cat# S3401, Sigma-Aldrich) supplemented with 2% 2-mercaptoethanol (Cat# 19-1335, Sigma-Aldrich), denatured by boiling, and resolved in polyacrylamide gels (8–15%) at 110 V for 2 h. For preparing multiple transmembrane proteins, i.e., GLAST, GLT-1, EAAT3, vGluT-1, GAT1, and GAT3, the samples were dissolved in sample buffer (Cat# S3401, Sigma-Aldrich) with the omission of 2-mercaptoethanol, and the boiling step was skipped to avoid protein aggregation. The separated proteins were transferred to PVDF membranes (Cat# IPVH00010, Merck-Millipore), blocked with 5% skim milk, and hybridized with proper primary antibodies for 16 h at 4°C. After washing, the membranes were subsequently hybridized with proper horseradish peroxidase (HRP)-conjugated secondary antibodies (goat anti-mouse IgG: Cat# 115-035-166; goat anti-rabbit IgG: Cat# 111-035-144, Jackson ImmunoResearch, West Grove, PA, USA). The bound antibodies were detected using an enhanced chemiluminescence detection kit (Cat# WBKLS0500, Merck-Millipore) and X-ray film (Cat# Super RX, Fujifilm, Tokyo, Japan). Relative protein expression was estimated by normalizing with the β-actin or α-tubulin level. The band densities were analyzed using ImageJ software (v2.0.0-rc-69/1.52p, U.S. National Institutes of Health, Bethesda, MD, USA). For re-probing, the membranes were incubated with a stripping buffer containing 2% SDS, 62.5 mM Tris, and 0.8% 2-mercaptoethanol for 20 min at 55°C to remove the bound antibodies. The dilution ratios of antibodies and the image exposure time were tested to confirm that the luminescence signals were within the linear range of detection. Antibodies used in this study are listed in **Supplementary Table S3**.

**Fluoro-Gold retrograde tracing**

One week before concluding the experiments, the mice were anesthetized with Zoletil 50 (75 mg/kg, i.p.; Virbac), mounted on a stereotaxic apparatus (Model: 51500, Stoelting, Wood Dale, IL, USA), and received infusions of Fluoro-Gold (FG, 5% dissolved in ultrapure water, Cat# sc-358883, Santa Cruz Biotechnology, Dallas, TX, USA) into their bilateral NAc (AP: +1.0 mm; ML: ±1.0 mm; DV: -4.6 mm from the bregma) at the rate of 0.005 µL/min via a 30G stainless steel needle connected to a syringe (Model: 80000, Hamilton, Reno, NV, USA) with a PE-10 tube (Cat# BTPE-10, Instech, Plymouth Meeting, PA, USA). The infusion rate was controlled by a microsyringe pump (Model: KDS 210, KD Scientific, Holliston, MA, USA). After completing the infusion, the needle was kept in place for 10 min before withdrawal to prevent reflux. The incisions were closed with Michel suture clips (Cat# BN507R, Aesculap, Center Valley, PA, USA). We compared the diffusion of various volumes of FG (i.e., 0.03, 0.05, and 0.08 µL) infused to the NAc and found that an infusion of 0.03 µL resulted in a more restricted diffusion pattern than the other two (**Supplementary Figure S1a**). Therefore, we infused 0.03 µL of FG to each side of the NAc to label the NAc-projecting neurons.

**Immunostaining**

Mice were anesthetized with Zoletil 50 (75 mg/kg, i.p.; Virbac) and transcardially perfused with chilled PBS (Cat# 10010023, Thermo Fisher Scientific) and 4% paraformaldehyde (Cat# 158127, Sigma-Aldrich) prepared in 0.1M phosphate buffer (Cat# P3619, Sigma-Aldrich). Their brains were removed, post-fixed with buffered 4% paraformaldehyde for 24 h, and dehydrated daily with gradually increasing concentrations (10, 20, 30, and 35%, twice per concentration) of sucrose (Cat# S9378, Sigma-Aldrich) solutions prepared in 0.1 M phosphate buffer (Cat# P3619, Sigma-Aldrich). The dehydrated brains were embedded in the cutting compound (Cat# 3801480, Leica Biosystems, Wetzlar, Hessen, Germany) and sliced into 25-µm coronal sections using a cryostat (Model: CM1950, Leica Biosystems).

Immunohistochemistry of FG and mCherry were performed to determine the spatial distributions of the NAc-projecting neurons in the brain of mice that received bilateral infusions of FG or rAAV into the NAc, respectively. The coronal brain sections were washed with PBS (Cat# 10010023, Thermo Fisher Scientific) containing 0.3% Triton X-100 (Cat# X100, Sigma-Aldrich) (PBST), incubated with PBST containing 3% H_2_O_2_ (Cat# H1009, Sigma-Aldrich) for 20 min at room temperature, blocked with 3% normal goat (Cat# S26-M, Sigma-Aldrich, used in staining FG) or donkey serum (Cat# S30-M, Sigma-Aldrich, used in staining mCherry) prepared in PBST for 1 h at room temperature, and probed with primary antibodies against FG (1: 500 dilution, Cat# AB153-I, Merck-Millipore) and mCherry (1: 200 dilution, Cat# AB0040-200, OriGene Technologies, Rockville, MD, USA) for 16 h at room temperature. After washing out unbound antibodies, the brain sections were incubated with HRP-conjugated secondary antibodies (for FG: 1:500 dilution, goat anti-rabbit IgG, Cat# 111-035-144; for mCherry: 1:500 dilution, donkey anti-goat IgG, Cat# 705-035-147, Jackson ImmunoResearch) for 2 h at room temperature, followed by PBST washes, and incubated with the chromogen, 3,3′-Diaminobenzidine (Cat# D12384, Sigma-Aldrich). After dehydration, sections were mounted with the xylene-based mounting medium (Cat# 3801730, Leica Biosystems). The FG-immunoreactive (FG^+^) and mCherry-immunoreactive (mCherry^+^) cells are shown in **Supplementary Figures S1c** and **S3a**.

Immunofluorescence was used to determine the expression levels of glutaminase and c-Fos in the mPFC, lateral and basolateral amygdala (BLA), and vHPC. The brain sections were washed and blocked as aforementioned and incubated with primary antibodies against glutaminase (1: 1,000 dilution, Cat# 12855-1-AP, Proteintech, IL, USA) and c-Fos (1: 1,000 dilution, Cat# ab190289, Abcam, Cambridge, UK) for 16 h at room temperature. Then, the brain sections were incubated with the Alexa Fluor-conjugated secondary antibodies (for glutaminase: 1:1,000 dilution, Alexa Fluor 488-conjugated goat anti-rabbit IgG, Cat# A-11034; for c-Fos: 1:1,000 dilution, Alexa Fluor 488- or Alexa Fluor 594-conjugated goat anti-rabbit IgG, Cat# A-11034 and A-11037, Thermo Fisher Scientific) for 2 h at room temperature, washed with PBST, and mounted in media with (Cat# ab104139, Abcam) or without (Cat# 00-4958-02, Thermo Fisher Scientific) DAPI. Omissions of primary antibodies were used for detecting nonspecific binding in both staining methods. Sections of the same experiment were stained together to minimize batch-to-batch variations.

**Image capture and analyses**

Immunohistochemical images were captured by an optical fluorescence microscope (Model: Axio Imager A1, Carl Zeiss, Oberkochen, Germany) equipped with a digital camera (Model: Axiocam 305 Color, Carl Zeiss). The immunofluorescent images used for quantifying the densities of NAc-projecting and c-Fos^+^ neurons were captured by the same system. A laser confocal microscope system (Model: FV1000, Olympus, Tokyo, Japan) was employed to determine the colocalization of glutaminase and retrograde tracer/reporter. ImageJ software (v2.0.0-rc-69/1.52p, U.S. National Institutes of Health) was used to merge the multiple color channels and analyze the parameters of interest.

The number of FG^+^, mCherry^+^, glutaminase^+^, and c-Fos^+^ cells were examined in the mPFC (AP: +2.0 ~ +1.6 mm from bregma), BLA (AP: -1.7 ~ -2.2 mm from bregma), and vHPC (AP: -3.2 ~ -3.6 mm from bregma). The sampling areas of these brain regions on coronal plans are indicated in **Fig. 2b**. A consistent background signal was set and applied to all analyses. Only those cells with over-threshold signals were counted as immunoreactive cells. The analyzers who counted the cells were blinded to the treatment. Five sections from each region of interest of each animal were determined, and the average was presented as a single data point.

**Details for chemogenetic inhibition**

Six weeks before ending the HFD, the mice were anesthetized with Zoletil 50 (75 mg/kg, i.p.; Virbac) and mounted on a stereotaxic apparatus (Model: 51500, Stoelting). Viral solutions contained pAAV-hSyn-hM4D_Gi_-mCherry (titer ≥ 7×10¹² vg/mL; Cat# 50475-AAVrg, Addgene) or pAAV-hSyn-mCherry (titer ≥ 7×10¹² vg/mL; Cat# 114472-AAVrg, Addgene) were bilaterally infused into NAc (0.6 µL/side, AP: +1.0 mm; ML: ±1.0 mm; DV: -4.6 mm from the bregma) of mice via a 30G stainless steel needle connected to a syringe (Model: 80000, Hamilton) at the rate of 0.02 µL/min. The infusion rate was controlled by a microsyringe pump (Model: KDS 210, KD Scientific). The injecting needle was withdrawn 10 min after the infusion was completed. The incisions were closed with Michel suture clips (Cat# BN507R, Aesculap).

Three days before ending the HFD, the mice were anesthetized with Zoletil 50 (75 mg/kg, i.p.; Virbac) and mounted on a stereotaxic apparatus (Model: 51500, Stoelting). Guiding cannulas (Cat# C315GS-4/SP 26G, P1 Technologies, Roanoke, VA, USA) were bilaterally implanted into the vHPC (AP: -3.2 mm; ML: ±2.8 mm; DV: -3.8 mm from the bregma) of the mouse and tightly fixed on its skull with dental adhesive resin cement (Model: Super-Bond, Sun Medical).

On the experiment day, CNO (Cat# 6329, Tocris Bioscience) was bilaterally infused into the vHPC (2 µg/µL dissolved in saline, 1 µL/side) of free-moving mice via stainless steel infusing needles (1 mm longer than the guiding cannula; Cat# C315IS-4/SP 33G, P1 Technologies) connected to a syringe (Model: 80365, Hamilton). The infusion rate (0.1 µL/min) was controlled by a microsyringe pump (Model: KDS 210, KD Scientific). The injecting needle was withdrawn 10 min after the infusion was completed. The mice were subjected to FST 30 min after the CNO infusion and sacrificed 120 min after completing the CNO infusions. Mice that received bilateral infusions of an equal volume of saline into the vHPC served as control. The procedures of euthanasia and brain sample collection were as described in those for immunostaining.

**Production of LVs**

The Lenti-X™ 293T cell line (Cat# 632180, Takara Bio, Shiga, Japan), a highly transfectable subclone of the human embryonic kidney HEK 293 cell line, was used to produce LVs. Cells were cultured with Dulbecco's Modified Eagle Medium (Cat# 11965092, Thermo Fisher Scientific) supplemented with 4 mM L-glutamine (Cat# 35050061, Thermo Fisher Scientific), 10% fetal bovine serum (Cat# TMS-013-BKR, Merck-Millipore), and penicillin-streptomycin (Cat# 15140122, Thermo Fisher Scientific) and maintained in a humidified atmosphere of 5% CO_2_ and 95% air at 37°C.

To enhance the tropism to astrocytes, the LVs were pseudotyped with the glycoprotein of Mokola virus ^1^. For each 6-cm dish of Lenti-X™ 293T cells, a 500-µL of Opti-MEM (Cat# 31985070, Thermo Fisher Scientific)-based transfection mixture containing 15 µL of TranIT-LT1 transfection reagent (Cat# MIR2300, Mirus Bio, Madison, WI, USA), 2.25 µg of pCMV-ΔR8.91 packaging plasmid (RNAi core in Academia Sinica, Nangang, Taipei, Taiwan), 0.25 µg of pHCMV-MokolaG plasmid (Cat# 15811, Addgene), and 2.5 µg of pLKO.1 shRNA-expressing plasmid (shLacZ: Cat# TRCN0000231722, target sequence: 5′-CGCGATCGTAATCACCCGAGT-3′; shGLAST: Cat# TRCN0000009923, target sequence: 5′-TGCCTATCCAGTCCAACGAAA-3′; shGLT-1: Cat# TRCN0000079845, target sequence: 5′-CGCACACAACTCTGTCGTAAT-3′, RNAi core in Academia Sinica) or protein-expressing plasmid (GFP: Cat# LV590; GLAST-GFP: Cat# LV478055; GLT-1-GFP: Cat# LV476303, Applied Biological Materials, Richmond, BC, Canada] was added and incubated for 18 h. Then, the medium containing transfection mixtures was replaced with 5 mL fresh growth medium supplemented with 1% (W/V) bovine serum albumin (BSA, Cat# A8806, Sigma-Aldrich). The conditioned media containing LV particles were harvested every day for 3 days, centrifuged at 3,000 × g for 15 min to remove cell debris, concentrated with virus precipitation reagent (Cat# LV825A-1, System Biosciences, Palo Alto, CA, USA), re-suspended in PBS (Cat# 10010023, Thermo Fisher Scientific), and stored at -80°C. Lenti-X GoStix Plus test (Cat# 631280, Takara Bio) was used to determine the titer of each LV solution. An LV with a known titer was used as a reference virus to calculate the actual titer of LVs. The final titers of the generated LVs were higher than 10^9^ IFU/mL.

**Intra-vHPC infusion of LVs**

To investigate the effects of knockdown of vHPC GLAST and GLT-1 on the NAc-vHPC glutamatergic transmission and the exhibition of depression-like behaviors, the naïve 8-week-old male C57BL/6N mice were infused with shRNA-expressing LVs into their bilateral vHPC for 4 weeks. To study the effects of restoring vHPC GLAST and GLT-1 on the HFD-induced deficits, the CD and HFD mice were bilaterally infused with GLAST- and GLT-1-expressing LVs into their vHPC 4 weeks before concluding the HFD.

On the surgery day, mice were anesthetized with Zoletil 50 (75 mg/kg, i.p.; Virbac) and mounted on a stereotaxic apparatus (Model: 51500, Stoelting). A total of 1.5 µL (for knockdown: 1.5 µL of LV expressing shLacZ or 0.75 µL of LV expressing shGLAST + 0.75 µL of LV expressing shGLT-1; for overexpression: 1.5 µL of LV expressing GFP or 0.75 µL of LV expressing GLAST;GFP + 0.75 µL of LV expressing GLT-1;GFP) of LV was infused in three locations (0.5 µL/site, AP: -3.1, -3.3, and -3.6 mm; ML: ±2.8 mm; DV: -4.8 mm from the bregma) in each vHPC. The viral solutions were infused via a 30G stainless steel needle connected to a syringe (Model: 80000, Hamilton) with a PE-10 tube (Cat# BTPE-10, Instech) at the rate of 0.05 µL/min. The infusion rate was controlled by a microsyringe pump (Model: KDS 210, KD Scientific). The needle was withdrawn 10 min after ending the infusion. The incisions were closed with Michel suture clips (Cat# BN507R, Aesculap). By comparing the temporal knockdown efficiencies of various shRNAs for each target, we adopted a recipe (i.e., #1 shRNA for GLAST and 3# shRNA for GLT-1, infection time: 4 weeks) for related experiments (**Supplementary Figures S6a** and **b**).

**Details for intra-vHPC infusion of riluzole**

Ten days before the end of the HFD feeding period, mice were anesthetized with Zoletil 50 (75 mg/kg, i.p.; Virbac) and mounted on a stereotaxic apparatus (Model: 51500, Stoelting). Guiding cannulas (Cat# C315GS-4/SP 26G, P1 Technologies) were bilaterally implanted into the vHPC (AP: -3.2 mm; ML: ±2.8 mm; DV: -3.8 mm from the bregma) and tightly fixed on the skull with dental adhesive resin cement (Model: Super-Bond, Sun Medical). After a 3-day recovery period, riluzole (RLZ, Cat# 1604337, Sigma-Aldrich) was bilaterally infused daily into the vHPC (0.5, 1, or 2 nmol dissolved in 0.5 µL artificial cerebrospinal fluid containing 1% DMSO/side) of free-moving mice via stainless steel infusing needles (1 mm longer than the guiding cannula; Cat# C315IS-4/SP 33G, P1 Technologies) connected to a syringe (Model: 80365, Hamilton) for 7 days. The infusion rate (0.05 µL/min) was controlled by a microsyringe pump (Model: KDS 210, KD Scientific). The injecting needle was withdrawn 10 min after the infusion was completed. Vehicle control mice received bilateral infusions of an equal volume of artificial cerebrospinal fluid containing 1% DMSO into the vHPC. In the groups of mice used for FG retrograde tracing, the bilateral intra-NAc infusion of FG was conducted at the same time as the bilateral intra-vHPC implantation of cannulas.

**Details for statistical analysis**

All numerical data are expressed as mean ± standard deviation. Statistical analyses and graph plotting were performed using the Prism software (v. 7.0a, GraphPad Software Inc., San Diego, CA, USA). Significance was set at *P* < 0.05. To faithfully report our results, we presented all acquired data points without removing any data. Our statistical analysis revealed a high effect size (**Supplementary Table S2**). For the estimation of sample size, we used MedCalc® software (version 20.109, MedCalc Software Ltd, Ostend, Belgium) with the following settings: type I error = 0.05, type II error = 0.20, ratio of sample sizes in group 1/group 2 = 0.8-1. Based on the means and standard deviations of CD and HFD groups (difference of means = 25.81, standard deviation in CD group = 18.84, standard deviation in HFD group = 19.96) (**Fig. 1k**), the minimal sample size for FST was 8-10. We followed this estimation to carry out subsequent studies. The D’Agostino-Pearson normality test was adopted to estimate the assumption of normality. For datasets with normal distributions, unpaired, two-tailed Student’s *t*-test was used to compare means in experiments with two groups, while one-way ANOVA followed by Tukey’s multiple comparisons was used to analyze differences between more than two independent groups. For non-normal distributed datasets, the Mann-Whitney *U* test was used for comparisons of two groups, and the Kruskal-Wallis test followed by Dunn’s multiple comparisons was used to analyze differences between more than two independent groups. Designs with two independent variables were analyzed by two-way ANOVA followed by Tukey’s multiple comparison tests if the main effects or interactions were significant. Repeated measures two-way ANOVA followed by Sidak’s multiple comparisons was used to analyze the body weights of mice. The area under the curve calculation analyzed the performance of IPGTT and IPITT. The effect size was measured with Cohen’s d value calculated by the following formula:

(mean of group A - mean of group B)/√[(standard deviation of group A2 + standard deviation of group B2)/2)

if the difference between the two groups was significant. The details of statistical analysis are described in **Supplementary Table S2**.

**2. Legends for Supplementary Figures**

**Supplementary Fig. S1 Four weeks of fluoxetine treatment alleviates HFD-induced exhibition of depression-like behaviors in mice**. **a** Results of SPT. n = 5 cages per group. **b** Results of FST. n = 10 mice per group. All data are represented as mean ± SD. n.s., not significant. See also Supplementary Table S1 and S2 for details of animal usage and statistical test results.

**Supplementary Fig. S2 Optimization of procedures of retrograde tracing using FG infused in the NAc of mice and determination its labelling patterns. a** Diffusing areas of FG at different administrated volumes around the infusing site (NAc), which was detected at 1 week after infusion. The outer yellow dash circle indicates the area of core of NAc and the inner white dash circle indicates the areas of anterior commissure. **b** Images of original fluorescence signal and immunohistochemistry signal of FG on the same brain section. The signals of these two pictures were highly similar to each other, validating the accuracy of antibody against FG used in this study. **c** Images of immunohistochemistry of FG showing the defined regions retrogradely traced by the intra-NAc infused FG in the mouse brain, including the mPFC, piriform area, thalamus, BLA, vHPC and VTA. **d** Confocal fluorescence images showing colocalizations of FG and glutaminase, a marker for glutamatergic neurons, in the mPFC, BLA, vHPC of mice received intra-NAc infusion of FG. Scale bar, 50 µm.

**Supplementary Fig. S3 Determination of labeling patterns of mCherry-expressing rAAV infused in the NAc of mice. a** Images of fluorescence signals of mCherry in the NAc, mPFC, BLA and vHPC of mice received intra-NAc infusion of rAAV expressing mCherry 6 weeks ago. **b** Images of immunohistochemistry of mCherry showing the defined regions retrogradely traced by the intra-NAc infused mCherry-expressing rAAV in the mouse brain, including the mPFC, piriform area, thalamus, BLA and vHPC. **c** Confocal fluorescence images showing colocalizations of mCherry and glutaminase, in the mPFC, BLA, vHPC of mice received intra-NAc infusion of mCherry-expressing rAAV. Scale bar, 50 µm.

**Supplementary Fig. S4 Detailed fluorescence images incorporating DAPI signals for the vHPC of mice used in chemogenetic inhibition and effects of chemogenetic inhibition of the NAc-projecting vHPC neurons on c-Fos expression in the NAc-projecting mPFC and BLA neurons. a** Detailed fluorescence images incorporating DAPI signals for the vHPC of mice subjected in experiments of chemogenetic inhibition. **b**-**d** Effects of chemogenetic inhibition of the NAc-projecting vHPC neurons on c-Fos expression in the NAc-projecting mPFC neurons in mice. **b** Representative fluorescence images for c-Fos (green), mCherry (red) and DAPI (blue). Scale bar, 100 µm. **c** Number of mCherry-expressing NAc-projecting mPFC neurons. **d** Fraction of c-Fos-expressing neurons in the mCherry-expressing ones. n = 17 mice in CD-Saline group; n = 17 mice in CD-CNO group; n = 20 mice in HFD-Saline group; n = 19 in HFD-CNO group. **e**-**g** Effects of chemogenetic inhibition of the NAc-projecting vHPC neurons on c-Fos expression in the NAc-projecting BLA neurons in mice. **e** Representative fluorescence images for c-Fos (green), mCherry (red) and DAPI (blue). Scale bar, 100 µm. **f** Number of mCherry-expressing NAc-projecting BLA neurons. **g** Fraction of c-Fos-expressing neurons in the mCherry-expressing ones. n = 17 mice in CD-Saline group; n = 17 mice in CD-CNO group; n = 20 mice in HFD-Saline group; n = 19 in HFD-CNO group. All data are represented as mean ± SD. n.s., not significant. See also Supplementary Table S1 and S2 for details of animal usage and statistical test results.

**Supplementary Fig. S5 Intra-vHPC infusion of CNO failed to silence the NAc-projecting vHPC glutamatergic neurons and alleviate depressive phenotype in HFD mice received intra-NAc infusion of rAAV lacking expression of hM4D_Gi_. a** Representative fluorescence images for c-Fos (green), mCherry (red) and DAPI (blue). Scale bar, 100 µm. **b** Number of mCherry-expressing NAc-projecting vHPC neurons. **c** Fraction of c-Fos-expressing neurons in the mCherry-expressing neurons. **d** Results of FST. **e** Body weights of mice before FST. n = 20 mice in HFD(+)-hM4D_Gi_-mCherry(+)-mCherry(-)-CNO(-) group; n = 19 mice in HFD(+)-hM4D_Gi_-mCherry(+)-mCherry(-)-CNO(+) group; n = 9 mice in HFD(+)-hM4D_Gi_-mCherry(-)-mCherry(+)-CNO(+) group. The datasets of HFD(+)-hM4D_Gi_-mCherry(+)-mCherry(-)-CNO(-) and HFD(+)-hM4D_Gi_-mCherry(+)-mCherry(-)-CNO(+) groups are the same as those of HFD-Saline and HFD-CNO groups shown in Figure 2, respectively. All data are represented as mean ± SD. n.s., not significant. See also Supplementary Table S1 and S2 for details of animal usage and statistical test results.

**Supplementary Fig. S6 HFD did not affect expression of GLAST and GLT-1 in the mPFC and BLA. a** Measurements of expression of GLAST in the mPFC of mice. Left panels show the representative micrographs for Western blots. Right panel shows quantitative results. **b** Measurements of expression of GLT-1 in the mPFC of mice. Left panels show the representative micrographs for Western blots. Right panel shows quantitative results. **c** Measurements of expression of GLAST in the BLA of mice. Left panels show the representative micrographs for Western blots. Right panel shows quantitative results. **d** Measurements of expression of GLT-1 in the BLA of mice. Left panels show the representative micrographs for Western blots. Right panel shows quantitative results. n = 10 mice per group. All data are represented as mean ± SD. n.s., not significant. See also Supplementary Table S1 and S2 for details of animal usage and statistical test results.

**Supplementary Fig. S7 Validations of procedures of LV production. a** Determinations of effects of pseudotyping [vesicular stomatitis virus (VSV) vs. Mokola virus] and infectious period on efficacy of LVs expressing shGLASTs with different targeting sequences. Relative expression of the vHPC GLAST was detected by Western blot. The red frame indicates the chosen conditions of production and infectious period of LV expressing shGLAST. n = 2 mice per group. **b** Determinations of effects of pseudotyping (VSV vs. Mokola virus) and infectious period on efficacy of LVs expressing shGLT-1s with different targeting sequences. Relative expression of the vHPC GLT-1 was detected by Western blot. The red frame indicates the chosen conditions of production and infectious period of LV expressing shGLT-1. n = 2 mice per group. **c** Fluorescence images of 293T cultures infected with high-titer LVs expressing GFP, GLAST:GFP, and GLT-1:GFP for 2 days. Scale bar, 100 µm. **d** Micrographs of Western blots for measurements of expression of GLAST and GLT-1 in the 293T cultures infected with high-titer LVs expressing GFP, GLAST:GFP, and GLT-1:GFP for 2 days. All data are represented as mean ± SD. See also Supplementary Table S1 and S2 for details of animal usage and statistical test results.

**Supplementary Fig. S8 Determining the effective dose for the intra-vHPC infusion of RLZ. a** Local GLAST levels after 7-day daily intra-vHPC infusions of RLZ at dosages of 0.5, 1, and 2 nmol/side. Upper panels show representative Western blots. Lower panel shows quantitative results. **b** Local GLT-1 levels after 7-day daily intra-vHPC infusions of RLZ at dosages of 0.5, 1, and 2 nmol/side. Upper panels show representative Western blots. Lower panel shows quantitative results. n = 5 mice per group. All data are represented as mean ± SD. n.s., not significant. See also Supplementary Table S1 and S2 for details of animal usage and statistical test results.

**Supplementary Fig. S9 Three weeks of intraperitoneal RLZ did not affect HFD-induced obesity or systemic insulin resistance in mice. a** Body weight of mice during the experimental period. n = 20 mice in CD-Veh group; n = 19 mice in CD-RLZ group; n = 20 mice in HFD-Veh group; n = 22 mice in HFD-RLZ group. **b**-**d** Measurements of **b** fasting blood glucose levels, **c** fasting plasma insulin levels, and **d** HOMA-IR index in mice. n = 11 mice in CD-Veh group; n = 10 mice in CD-RLZ group; n = 11 mice in HFD-Veh group; n = 13 mice in HFD-RLZ group. **e** and **f** Results of IPGTT. **e** Blood glucose levels during IPGTT in mice. **f** AUC of IPGTT results. n = 11 mice in CD-Veh group; n = 10 mice in CD-RLZ group; n = 11 mice in HFD-Veh group; n = 13 mice in HFD-RLZ group. **g** and **h** Results of IPITT. **g** Blood glucose levels during IPITT in mice, and **h** AUC of IPITT results. n = 11 mice in CD-Veh group; n = 10 mice in CD-RLZ group; n = 11 mice in HFD-Veh group; n = 13 mice in HFD-RLZ group. All data are expressed as mean ± SD. n.s., not significant. See also Supplementary Table S1 and S2 for details of animal usage and statistical test results.

**Supplementary Fig. S10 Seven days of intra-vHPC RLZ did not affect HFD-induced obesity or systemic insulin resistance in mice**. **a** Body weight of mice during the experimental period. n = 20 mice per group. **b-d** Measurements of **b** fasting blood glucose levels, **c** fasting plasma insulin levels, and **d** HOMA-IR index in mice. n = 10 mice per group. **e** and **f** Results of IPGTT. **e** Blood glucose levels during IPGTT in mice. **f** AUC of IPGTT results. n = 10 mice per group. **g** and h Results of IPITT. **g** Blood glucose levels during IPITT in mice, and **h** AUC of IPITT results. n = 10 mice per group. All data are expressed as mean ± SD. n.s., not significant. See also Supplementary Table S1 and S2 for details of animal usage and statistical test results.

**3. Supplementary Table S3**

Supplementary Table S3 Information of antibodies used in this study

| **Antibodies** | **Source** | **Identifier** |
| --- | --- | --- |
| Rabbit polyclonal anti-glutamine synthetase (GS) | Proteintech | Cat#11037-2-AP; RRID: AB_2110650 |
| Rabbit monoclonal anti-glutamate/aspartate transporter (GLAST) | Cell Signaling Technology | Cat#5684S; RRID: AB_10695722 |
| Rabbit polyclonal anti-Glutamate transporter-1 (GLT-1) | Cell Signaling Technology | Cat#3838S; RRID: AB_2190743 |
| Rabbit monoclonal anti- excitatory amino acid transporter 3 (EAAT3) | Cell Signaling Technology | Cat#14501S; RRID: AB_2798499 |
| Rabbit monoclonal anti-AMPA receptor A1 (GluA1) | Cell Signaling Technology | Cat#13185S; RRID: AB_2732897 |
| Rabbit monoclonal anti-AMPA receptor A2 (GluA2) | Cell Signaling Technology | Cat#13607S; RRID: AB_2650557 |
| Rabbit monoclonal anti-AMPA receptor A3 (GluA3) | Cell Signaling Technology | Cat#4676S; RRID: AB_10547136 |
| Rabbit monoclonal anti-AMPA receptor A4 (GluA4) | Cell Signaling Technology | Cat#8070S; RRID: AB_10829469 |
| Rabbit monoclonal anti-NMDA Receptor 1 (GluN1) | Cell Signaling Technology | Cat#5704S; RRID: AB_1904067 |
| Rabbit polyclonal anti-NMDA Receptor 2A (GluN2A) | Cell Signaling Technology | Cat#4205S; RRID: AB_2112295 |
| Rabbit monoclonal anti-NMDA Receptor 2B (GluN2B) | Cell Signaling Technology | Cat#4212S; RRID: AB_2112463 |
| Rabbit polyclonal anti-Fluorescent Gold (Fluoro-Gold, FG) | Merck-Millipore | Cat#AB153-I; RRID: AB_2632408 |
| Rabbit polyclonal anti-glutaminase | Proteintech | Cat#12855-1-AP; RRID: AB_2110381 |
| Rabbit polyclonal anti-c-Fos | Abcam | Cat#ab190289; RRID: AB_2737414 |
| Goat polyclonal anti-mCherry | OriGene | Cat#AB0040-200; RRID: AB_2333092 |
| Mouse monoclonal anti-vesicular glutamate transporter-1 (vGluT-1) | Atlas Antibodies | Cat# AMAb91041; RRID: AB_2665777 |
| Rabbit monoclonal anti-glutamate decarboxylase 1 (GAD1, GAD67) | Cell Signaling Technology | Cat#41318S; RRID: AB_2799198 |
| Rabbit monoclonal anti-glutamate decarboxylase 2 (GAD2, GAD65) | Cell Signaling Technology | Cat#5843S; RRID: AB_10835855 |
| Rabbit polyclonal anti-GABAA Receptor α1 (GABARA1) | Sigma-Aldrich | Cat#06-868; RRID: AB_310272 |
| Mouse monoclonal anti-gephyrin | Santa Cruz Biotechnology | Cat#sc-25311; RRID: AB_627670 |
| Rabbit polyclonal anti-GABA transporter 1 (GAT1) | Atlas Antibodies | Cat#HPA013341; RRID: AB_1849509 |
| Rabbit polyclonal anti-GABA transporter 3 (GAT3) | Atlas Antibodies | Cat#HPA037981; RRID: AB_10671383 |
| Mouse monoclonal anti-β-actin | Sigma-Aldrich | Cat#A5441; RRID: AB_476744 |
| Mouse monoclonal anti-α-tubulin | Sigma-Aldrich | Cat#T9026; RRID: AB_477593 |
| HRP-conjugated goat anti-mouse IgG | Jackson ImmunoResearch | Cat#115-035-166; RRID: AB_2338511 |
| HRP-conjugated goat anti-rabbit IgG | Jackson ImmunoResearch | Cat#111-035-144; RRID: AB_2307391 |
| HRP-conjugated donkey anti-goat IgG | Jackson ImmunoResearch | Cat#705-035-147; RRID: AB_2313587 |
| Alexa Fluor 488-conjugated goat anti-rabbit IgG | Thermo Fisher Scientific | Cat#A-11034; RRID: AB_2576217 |
| Alexa Fluor 594-conjugated goat anti-rabbit IgG | Thermo Fisher Scientific | Cat#A-11037; RRID: AB_2534095 |

**4. Data and Resource Availability**

**Data and Resource Availability**

This study did not generate new unique reagents and codes. All datasets generated or analyzed in this study were included in the published article. Detailed datasets supporting the current study are available from the corresponding author upon request. Further information and requests for resources and reagents should be directed to and will be fulfilled by the corresponding author.

**Corresponding author**: Yu-Min Kuo (kuoym@mail.ncku.edu.tw).

**Supplemental References**

1. Delzor A, Escartin C, Deglon N. Lentiviral vectors: a powerful tool to target astrocytes in vivo. *Curr Drug Targets* 2013; **14**(11)**:** 1336-1346.
