# Supplementary figures and images for: High-fat diet induces depression-like phenotype via astrocyte-mediated hyperactivation of ventral hippocampal glutamatergic afferents to the nucleus accumbens
Source: Mol Psychiatry. 2022 Sep 30;27(11):4372–84. doi: 10.1038/s41380-022-01787-1 (PMC9734059; doi:10.1038/s41380-022-01787-1)

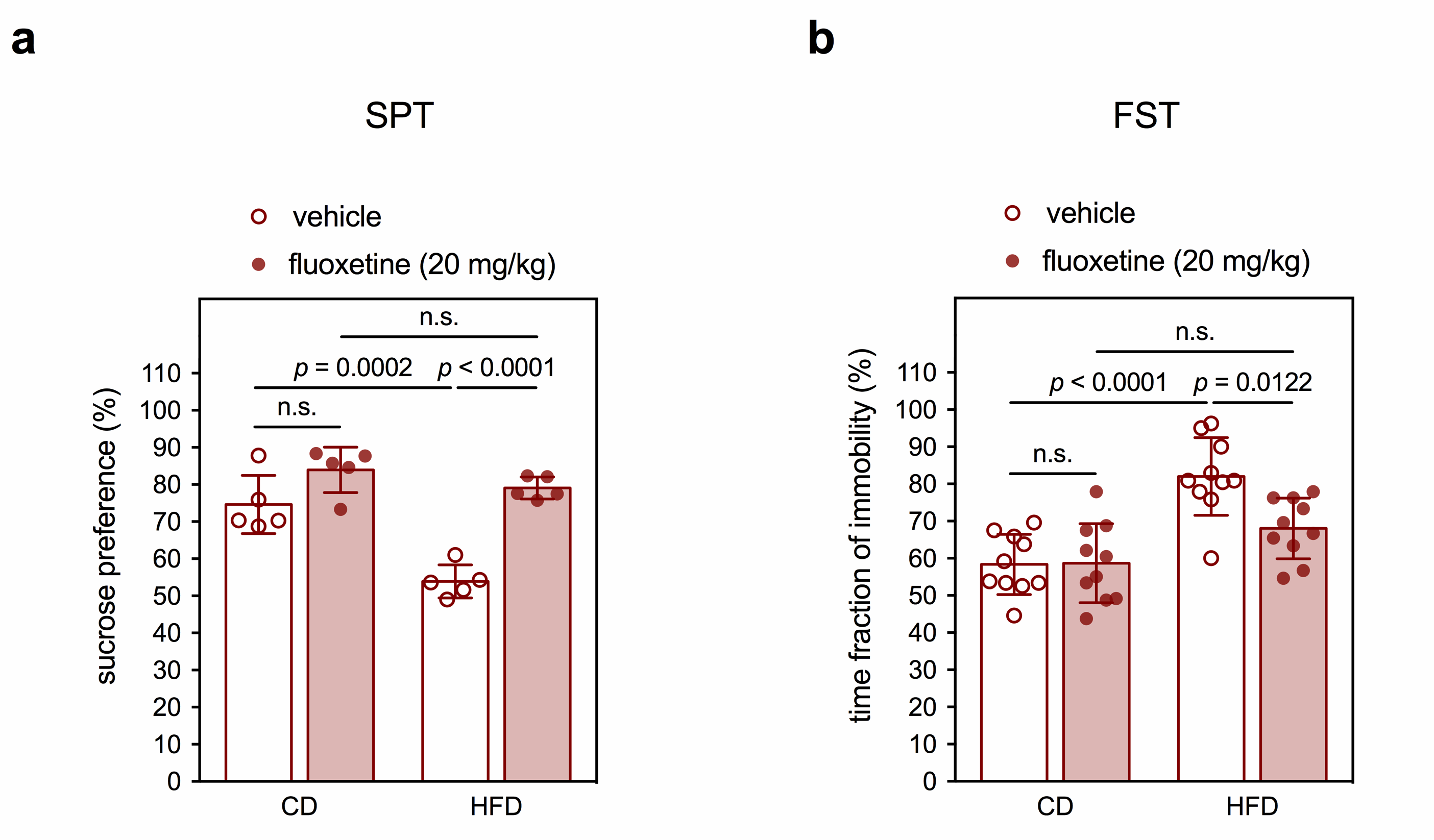

Supplement: Supplementary file 2 — Supplementary Figure S1 [file 41380_2022_1787_MOESM2_ESM.tif]

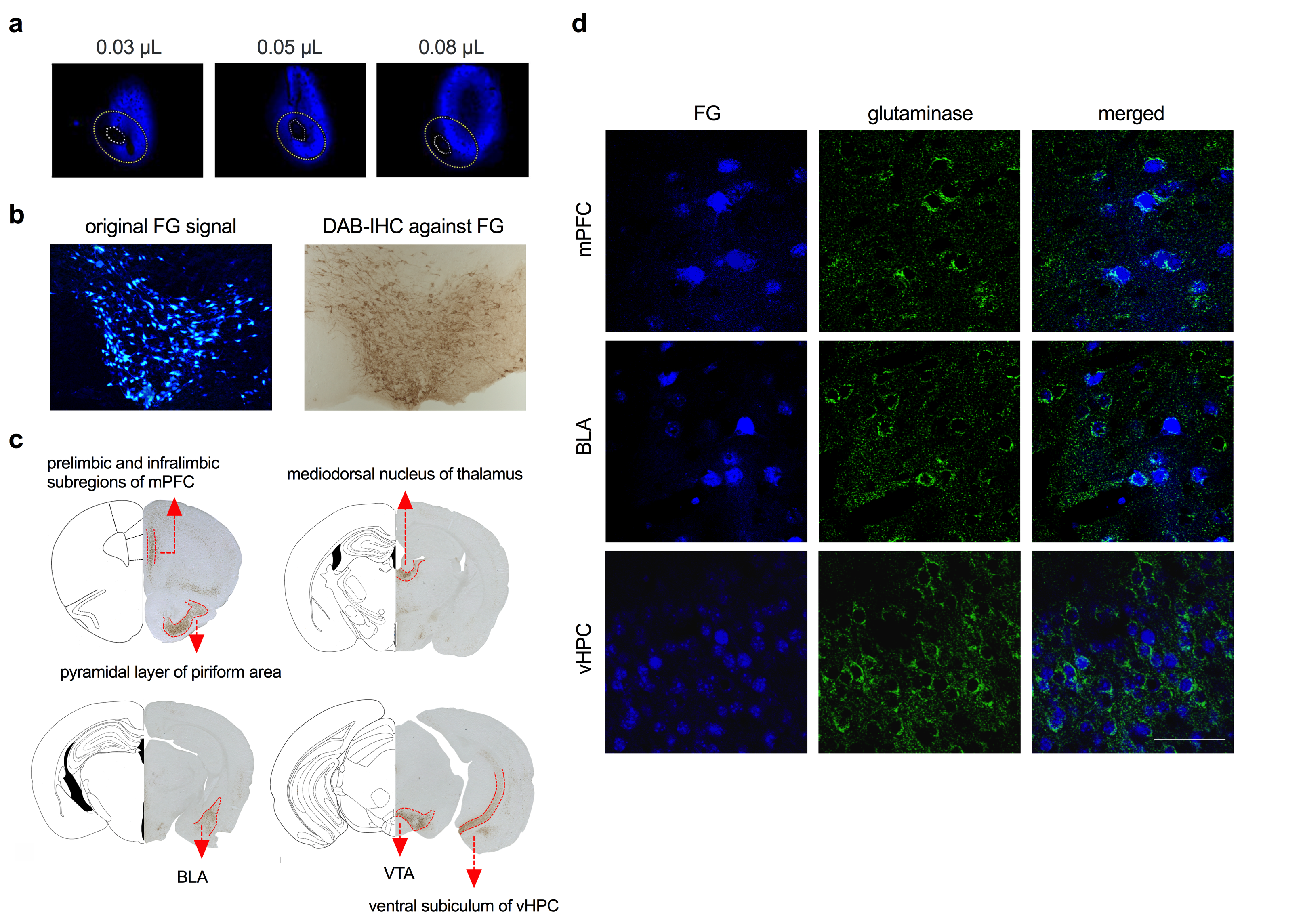

Supplement: Supplementary file 3 — Supplementary Figure S2 [file 41380_2022_1787_MOESM3_ESM.tif]

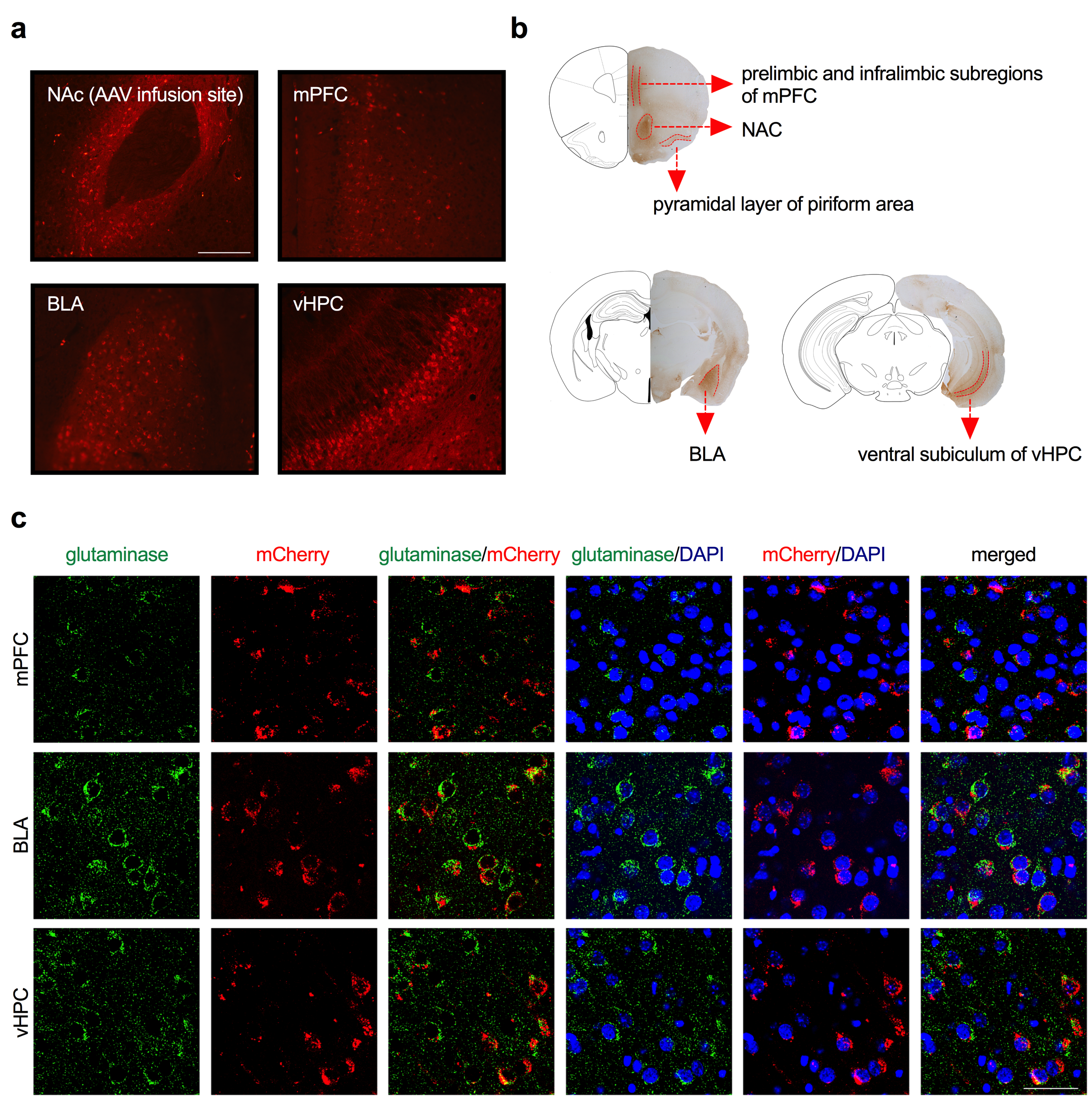

Supplement: Supplementary file 4 — Supplementary Figure S3 [file 41380_2022_1787_MOESM4_ESM.tif]

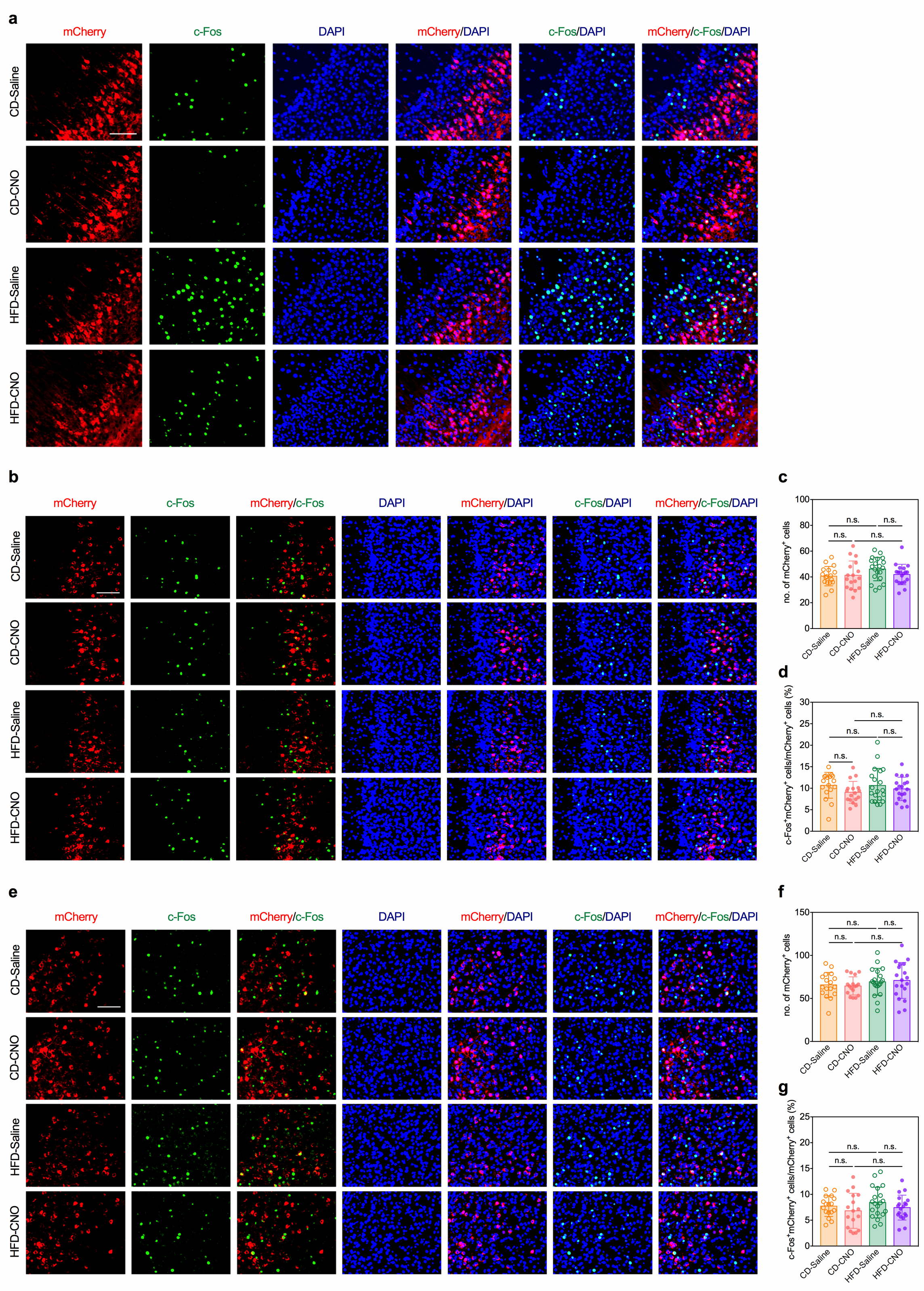

Supplement: Supplementary file 5 — Supplementary Figure S4 [file 41380_2022_1787_MOESM5_ESM.tif]

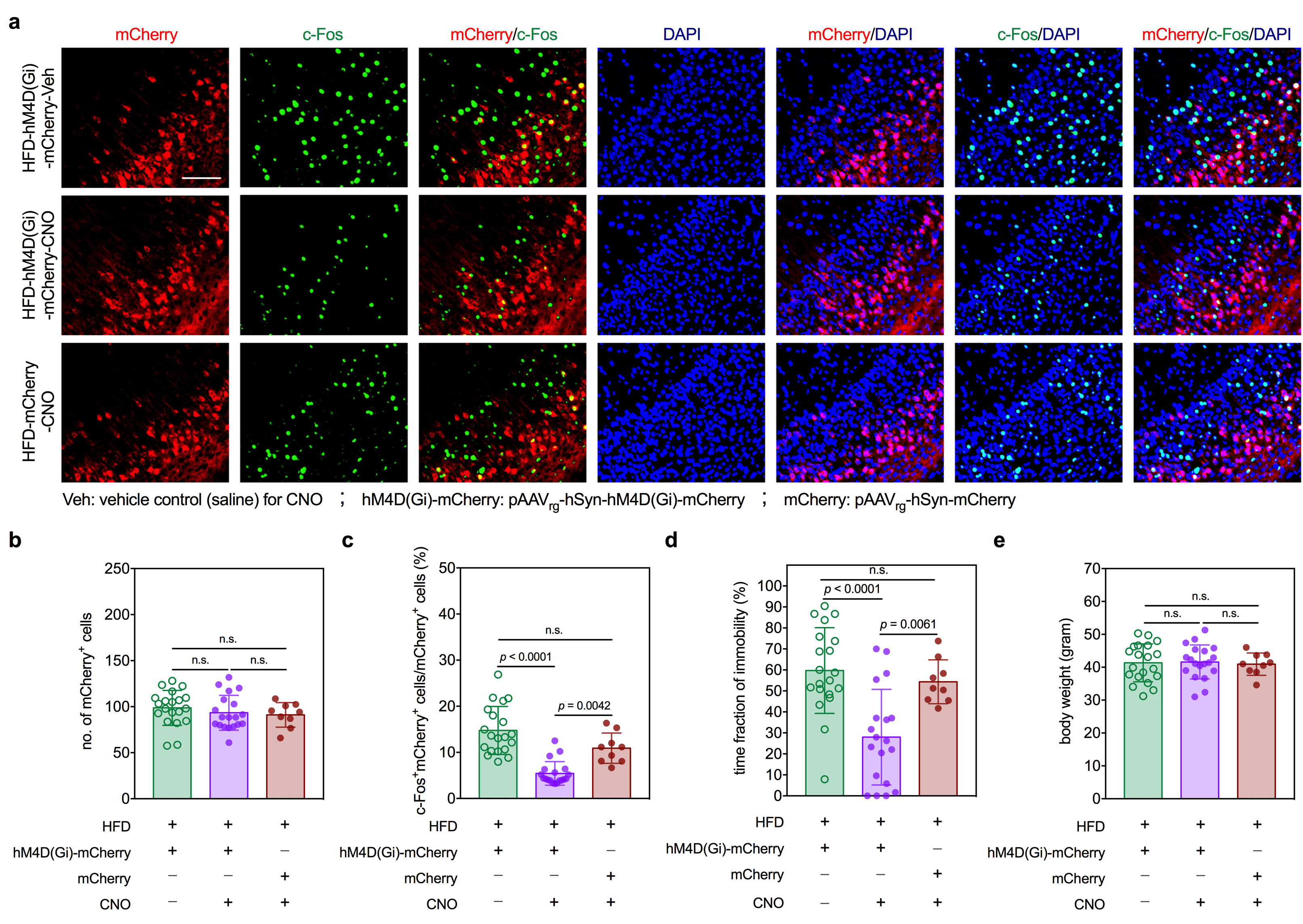

Supplement: Supplementary file 6 — Supplementary Figure S5 [file 41380_2022_1787_MOESM6_ESM.tif]

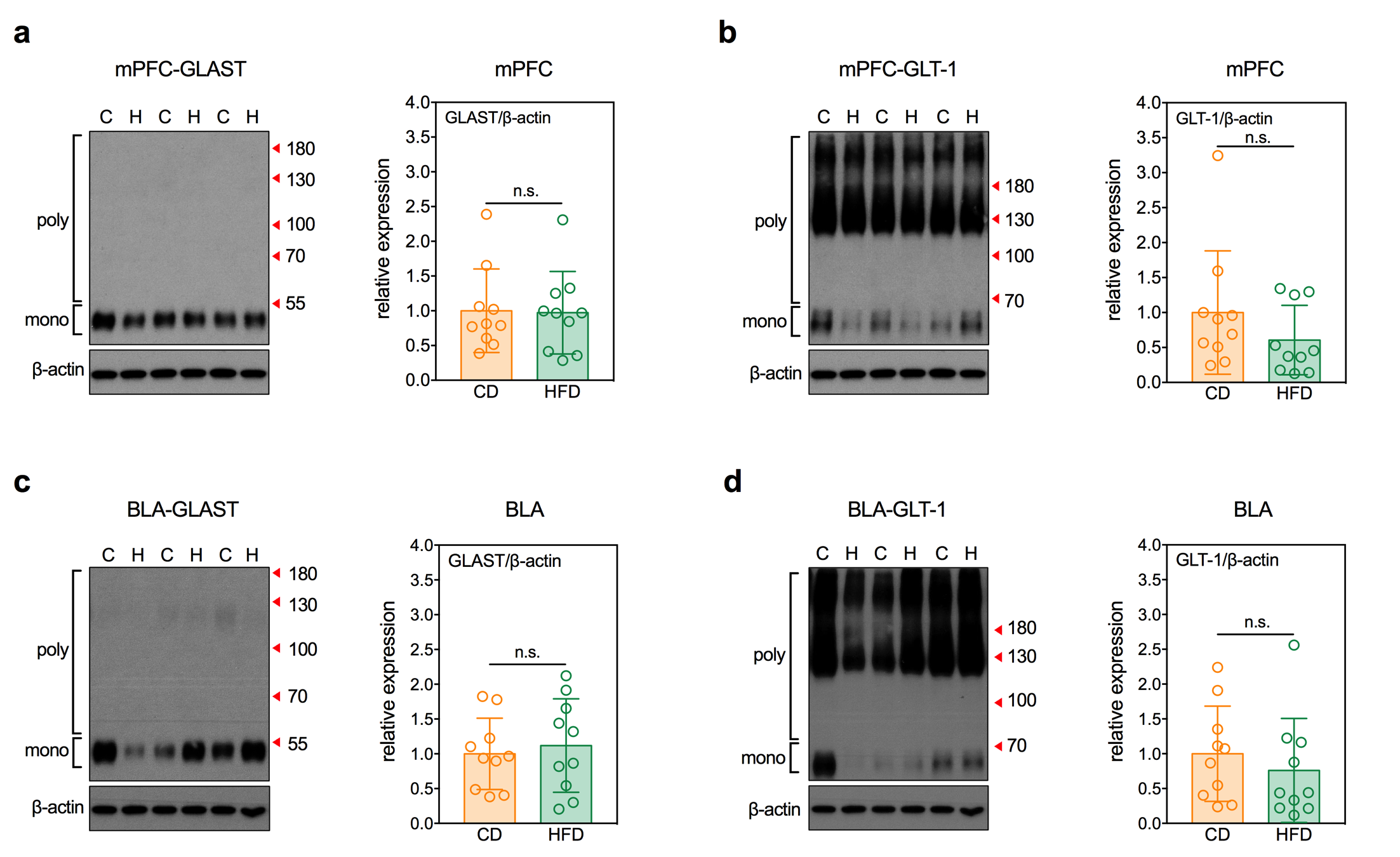

Supplement: Supplementary file 7 — Supplementary Figure S6 [file 41380_2022_1787_MOESM7_ESM.tif]

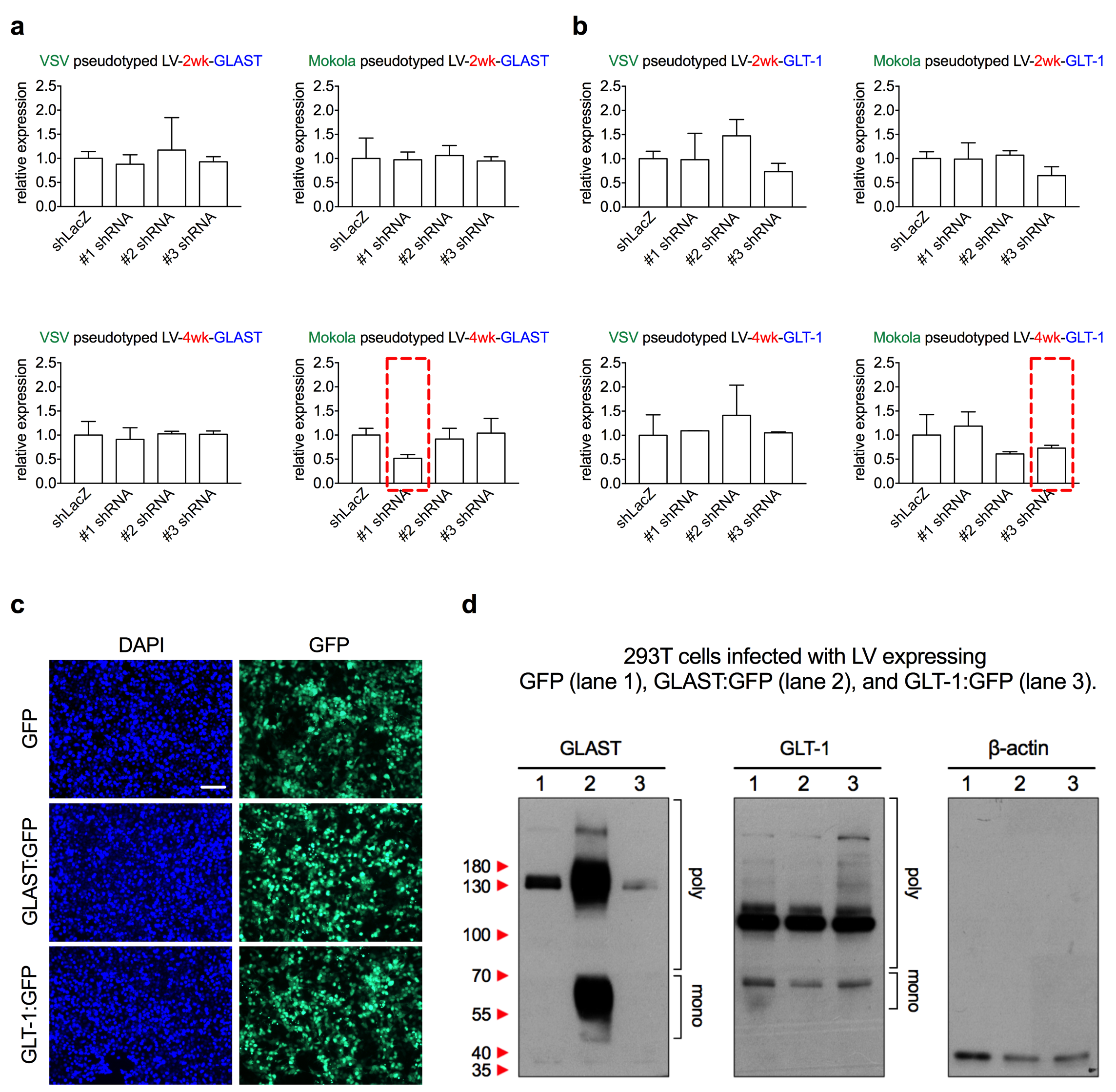

Supplement: Supplementary file 8 — Supplementary Figure S7 [file 41380_2022_1787_MOESM8_ESM.tif]

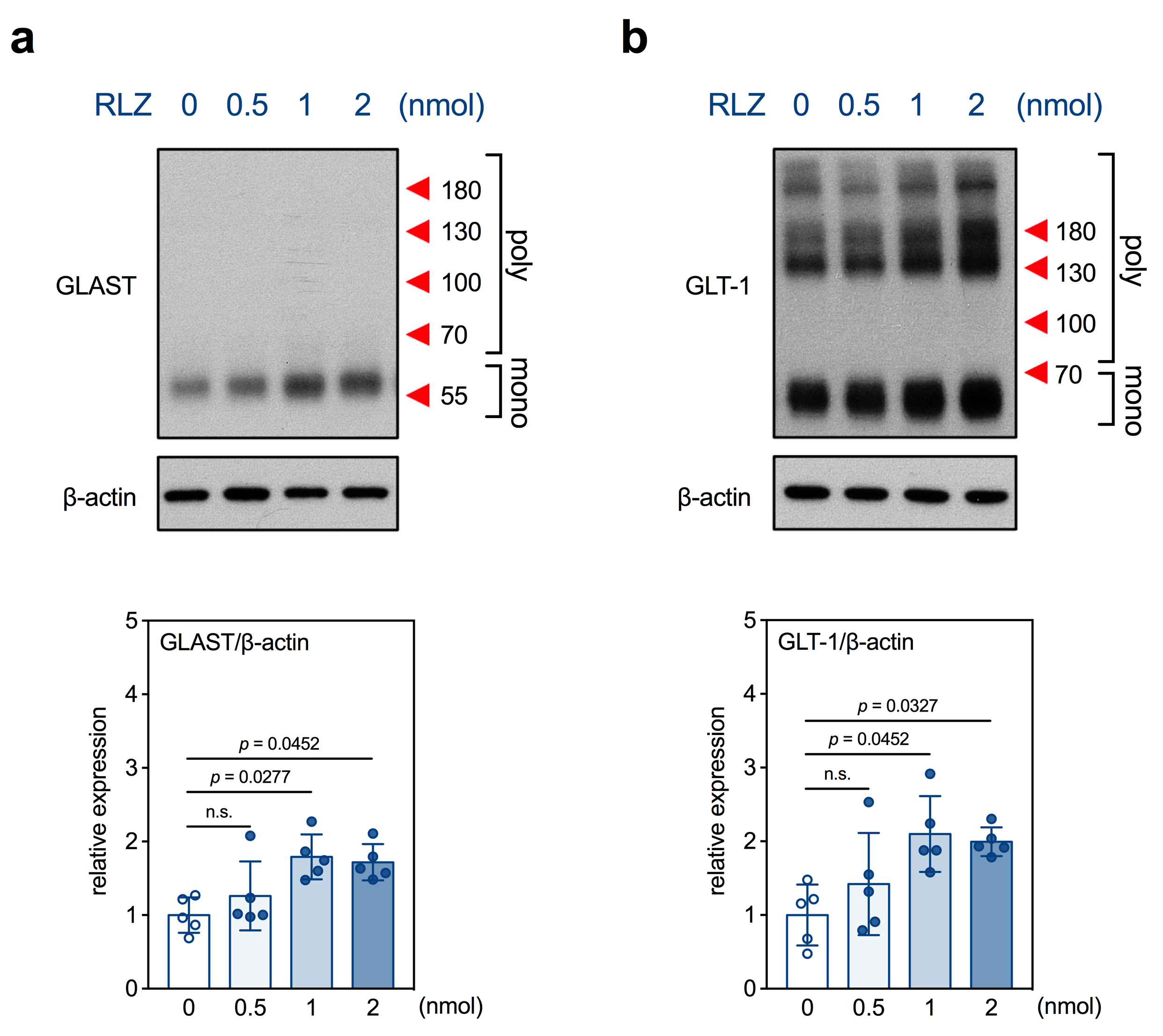

Supplement: Supplementary file 9 — Supplementary Figure S8 [file 41380_2022_1787_MOESM9_ESM.tif]

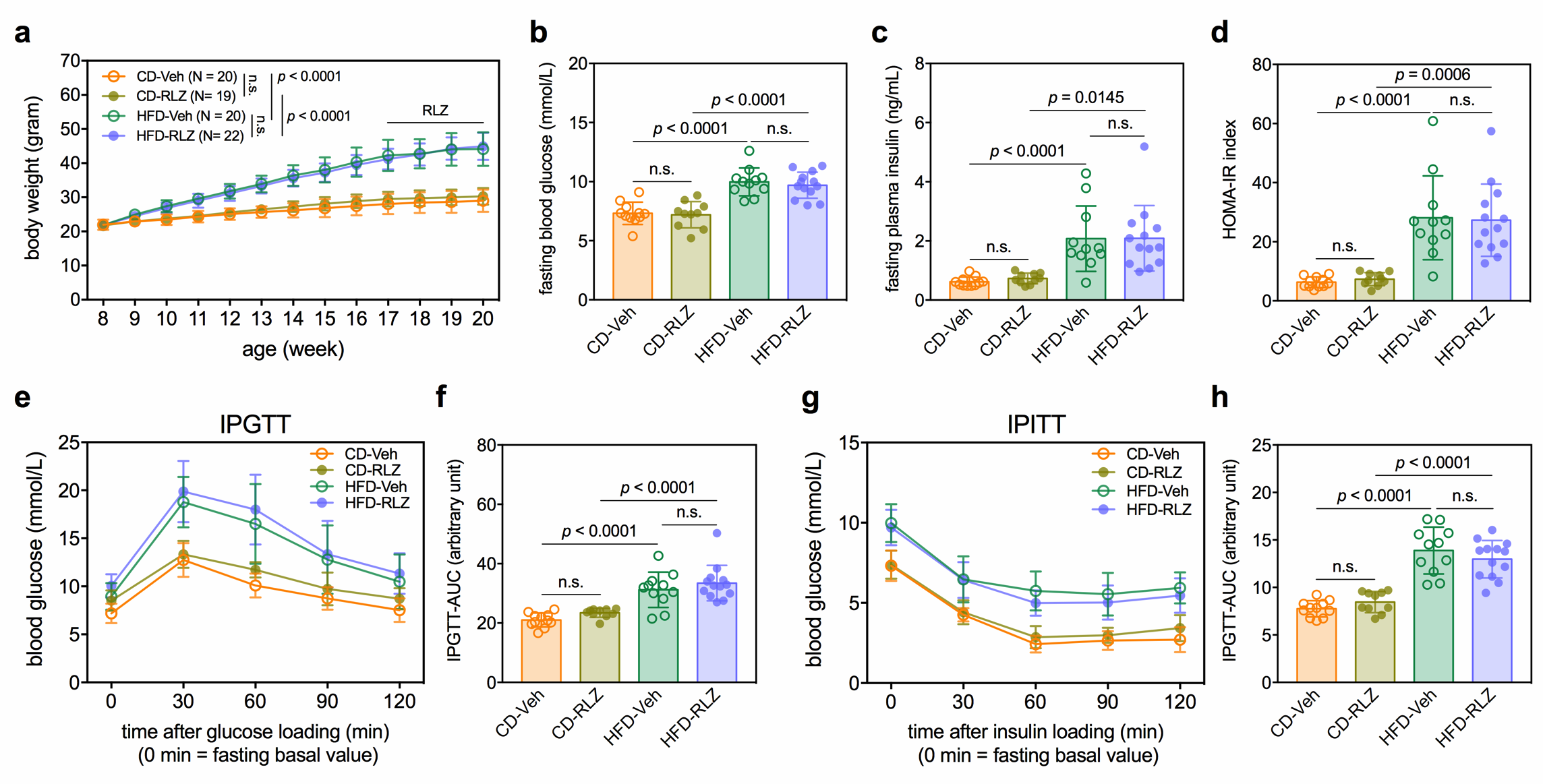

Supplement: Supplementary file 10 — Supplementary Figure S9 [file 41380_2022_1787_MOESM10_ESM.tif]

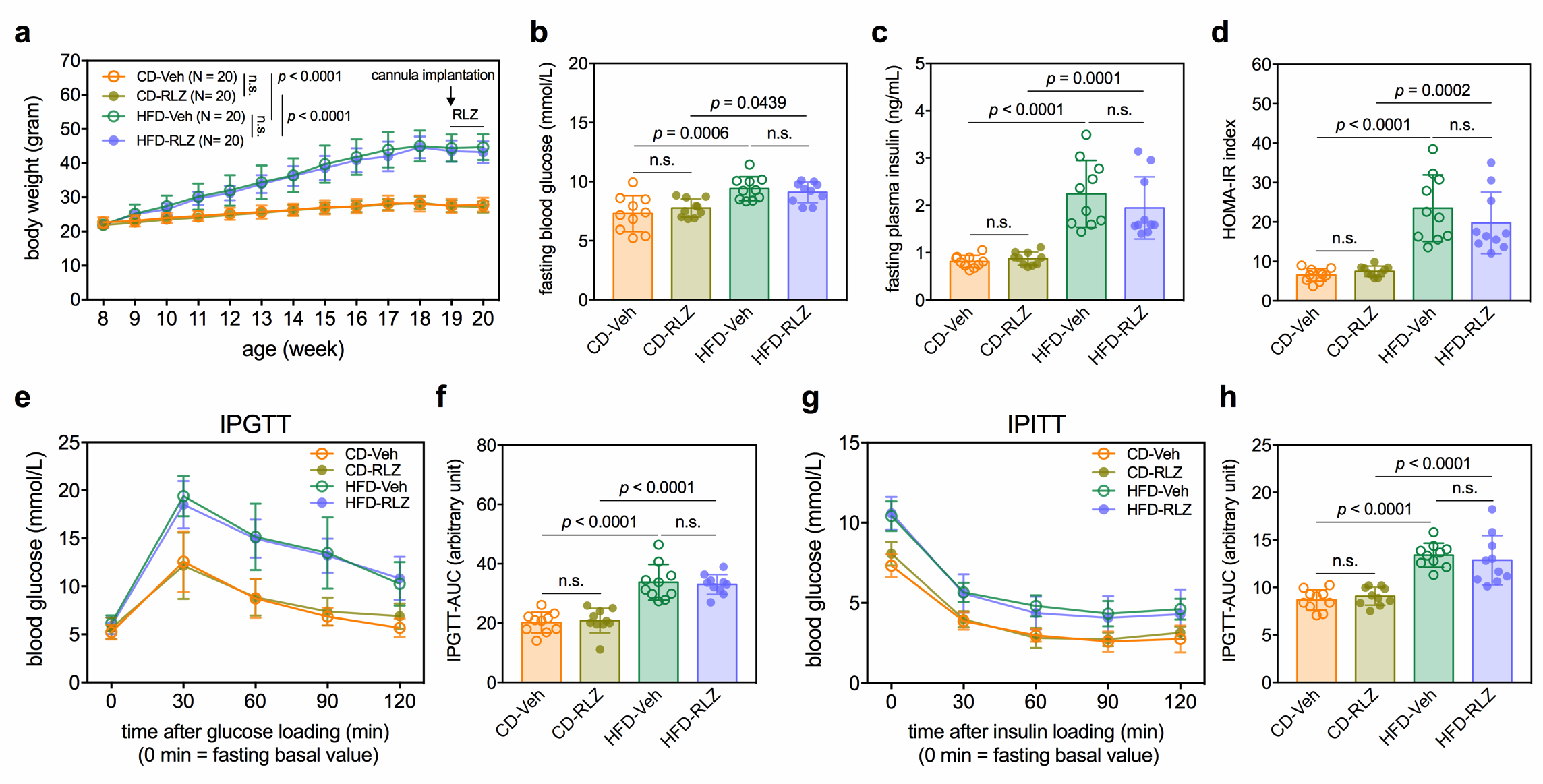

Supplement: Supplementary file 11 — Supplementary Figure S10 [file 41380_2022_1787_MOESM11_ESM.tif]
